# Efficient OLEDs Fabricated by Solution Process Based on Carbazole and Thienopyrrolediones Derivatives

**DOI:** 10.3390/molecules23020280

**Published:** 2018-01-30

**Authors:** Luis-Abraham Lozano-Hernández, José-Luis Maldonado, Cesar Garcias-Morales, Arian Espinosa Roa, Oracio Barbosa-García, Mario Rodríguez, Enrique Pérez-Gutiérrez

**Affiliations:** 1 Research Group of Optical Properties of Materials (GPOM), Centro de Investigaciones en Óptica, 37000 León, Guanajuato, Mexico; luislozano@cio.mx (L.-A.L.-H.); cgarcias@uadec.edu.mx (C.G.-M.); arian.espinosa@ciqa.edu.mx (A.E.R.); barbosag@cio.mx (O.B.-G.); mrodri@cio.mx (M.R.); 2 Departamento de Química Orgánica, Facultad de Ciencias Químicas, Universidad Autónoma de Coahuila, 25280 Saltillo, Coahuila, Mexico; 3 CONACYT-Laboratorio de Polímeros, Centro de Química, Instituto de Ciencias, Benemérita Universidad Autónoma de Puebla (BUAP), Complejo de Ciencias, ICUAP, 72570 Puebla, Puebla, Mexico; eperez@conacyt.mx

**Keywords:** OLEDs, small organic molecules, carbazole, thienopyrroledione, solution process

## Abstract

Four low molecular weight compounds—three of them new, two of them with carbazole (Cz) as functional group and the other two with thienopyrroledione (TPD) group—were used as emitting materials in organic light emitting diodes (OLEDs). Devices were fabricated with the configuration ITO/PEDOT:PSS/emitting material/LiF/Al. The hole injector layer (HIL) and the emitting sheet were deposited by spin coating; LiF and Al were thermally evaporated. OLEDs based on carbazole derivatives show luminances up to 4130 cd/m^2^, large current efficiencies about 20 cd/A and, cautiously, a very impressive External Quantum Efficiency (EQE) up to 9.5%, with electroluminescence peaks located around 490 nm (greenish blue region). Whereas, devices manufactured with TPD derivatives, present luminance up to 1729 cd/m^2^, current efficiencies about 4.5 cd/A and EQE of 1.5%. These results are very competitive regarding previous reported materials/devices.

## 1. Introduction

Since the manufacture of the first organic light emitting diodes (OLED) by Tang and Van Slyke [1], its study, development and maturity has intensified. The red, green and blue OLEDs (RGB) have great importance for commercial applications, however for the blue OLEDs, intense research is taking place because their efficiencies and lifetimes are lower than those for red and green devices [2]. The performance of OLEDs in general vary in terms of their efficiency values, for example, devices that emit in the wavelength range of 610–760 nm (red) typically have current efficiencies of 15 cd/A at luminances of 500 $\mathrm{cd}/m^{2}$, devices emitting in the range 500–570 nm (green) have current efficiencies of ~30 cd/A at larger luminance values (at 1000 $\mathrm{cd}/m^{2}$) and devices that emit in the range of 450–500 nm (blue or greenish blue) reach 19 cd/A at smaller luminance (at 100 cd/m^2^) [3]. Also, there are remarkable differences in their typical OLEDs lifetime values, for instance, for red-emitting devices lifetime is about 22,000 h (at 500 cd/m^2^), for the green-emitting devices about 20,000 h (at 1000 cd/m^2^) and, for blue-emitting devices: <1000 h (at 200 cd/m^2^) [3]; for this latter case, because blue emissive materials possess a large band gap, the charge injection is more difficult [4].

On the other hand, unlike polymers that form films with low RMS roughness by using the spin-coating technique, organic molecules of low molecular weight (SMs), usually form films with many defects (high RMS roughness), for this reason, they typically are deposited by evaporation processes for film formation, further; in several cases they do not have good solubility to be used under wet methods. However, for SMs with good solubility, there are some advantages such as acceptable film roughness and better molecular packing [5,6]. Therefore, the OLEDs assembly with SMs fabricated by solution methods is being investigated exhaustively since polymers, which are highly processable by wet methods (spin coating, blade coating, inkjet, gravure printing, slot die coating, etc.), are attractive because they can be manufactured in large area, may be light, flexible, self-emitters, have a low power consumption, high speed video rate, intense colors, high contrast and possess a potential lower cost when compared to evaporation methods (roll-to-roll process is other useful film fabrication technique) [7,8]. Moreover, SMs are also attractive for having a well-defined molecular structure that provides greater reproducibility in their synthesis [9], besides they are able of possessing a higher purity than polymers [10]. Thus, one of the most important factors to improve OLEDs efficiency is the design and synthesis of organic materials for the emitting layer [11]; both polymers as well as SMs play a crucial role for this technology. However, solution processing of SMs for optoelectronic devices, including OLEDs, tends to have some problems such as strong molecular aggregation, but at the same time, these drawbacks are also an area of great interest [12].

The carbazole unit is considered a virtuous building block for SMs with optoelectronics properties because of its good transportation of holes, amorphous film-forming properties, high triplet energy and good thermal stability [13]. Many organic materials for the fabrication of blue (or greenish blue) OLEDs are carbazole derivatives, which are used mainly as host materials [14,15,16,17,18,19,20,21,22,23,24] and in other cases, as emissive layer (EML) [25,26,27,28,29,30,31,32,33,34]. Previous studies of OLEDs fabrication based on carbazole derivatives have highlighted the great potential of them [35], for instance, S. Wang et al. [21] showed the use of G3MP, a carbazole derivative, as host in the fabrication of OLEDs with ITO/PEDOT:PSS/G3MP:G0/SPPO13/LiF/Al architecture, reporting high current efficiencies up to 54 cd/A and maximum luminances of $35,816\;\mathrm{cd}/m^{2}$ (at 18 V); M. Zhang et al. [36] reported OLEDs fabricated through solution process using TCBzC, a carbazole derivative, as emissive layer under the configuration ITO/PEDOT:PSS/TCBzC/TPBi/LiF/Al with a turn on voltage of 2.5 V, maximum luminances of 9226 cd/m^2^ and current efficiencies up to31.6 cd/A. Futhermore, P. Kotchapradist et al. [32] described OLEDs fabricated using CP3 as EML deposited by evaporation with a more robust configuration ITO/PEDOT:PSS/TPD/CP3/BCP/LiF/Al, with a turn on voltage of 2.7 V, maximum luminance of 244242 cd/m^2^ (at 9 V), with current efficiencies up to 6.9 cd/A and emitting at a wavelength of 501 nm, also they fabricated OLEDs with the same material but deposited by spin coating under the mentioned architecture with a turn on voltage of 3 V, reaching maximum luminance of 8235 cd/m^2^ (at 8.8 V) and a maximum current efficiency of 2.5 cd/A. C. Chan et al. [37] reported blue OLEDs (electroluminescence in λ_peak_ = 492 nm) with the ITO/LEL/TPBi/LiF/Al architecture (device III, no. 2), with a turn on voltage of 4.6 V, maximum luminance of 4390 cd/m^2^, current and luminous efficiencies of 1 cd/A and 0.5 lm/W, respectively. Likewise, J. Li et al. [38] fabricated OLEDs under the ITO/TPE-DFCz/TPBi/LiF architecture whose emissive layer (TPE-DFCz) is a carbazole derivative, with an emission wavelength of 500 nm, with a turn on voltage of 5.4 V, a maximum luminance of 3200 cd/m^2^ and a current efficiency of 1.2 cd/A.

Motivated by the large number of carbazole derivatives used for OLEDs [14,15,16,17,18,19,20,21,22,23,24,25,26,27,28,29,30,31,32,33,34], our group has synthesized two simple new carbazole derivatives (with dipolar electronic structure) to be used as emissive layer in the fabrication of greenish-blue OLEDs: (*E*)-3-(4-(9*H*-carbazol-9-yl)phenyl)-2-(thiophen-2-yl)acrylonitrile (CZ-2) and (*Z*)-3-(4-(9*H*-carbazol-9-yl)phenyl)-2-(4-bromophenyl)acrylonitrile (CZ-1). Such compounds have good solubility, good film formation by solution process, as well as they are of an easy, economical and fast synthesis. With these new carbazole derivatives, the fabricated OLEDs achieved maximum luminances of 4104 and 4130 cd/m^2^ for CZ-2 and CZ-1, respectively. These same OLEDs based on CZ-2 and CZ-1 compounds, showed also excellent maximum current efficiencies of 20.2 and 19.3 cd/A, with electroluminescence peaks located at 488 and 492 nm, respectively. Further, two other materials were also synthesized and used as emitters, thienopyrroledine (TPD) derivatives (with quadrupolar structure): 1,3-bis(4-(diphenylamino)phenyl-5-(2-ethylhexyl)-4*H*-thieno[3,4-*c*]pyrrole-4,6(5*H*)-dione (MOC-1); this compound was previously reported by C. Garcias et al. [39], and the new compound 1,3-bis(benzo[*b*]thiophen-2-yl)-5-(2-ethylhexyl)-4*H*-thieno[3,4-*c*]pyrrole-4,6(5*H*)-dione (MOC-16). With these TPD derivatives, luminances up to 651 and 1729 cd/m^2^ for OLEDs based on MOC-1 and MOC-16, respectively, were obtained. The electroluminescence peaks of MOC-1 and MOC-16 were located at 564 and 567 nm, respectively, while their maximum current efficiencies were 4.5 and 0.61 cd/A, respectively. Previous reports of TPD derivatives are mainly for the manufacturing of organic photovoltaic devices (OPVs) [39,40,41,42] and not as EML for OLEDs devices. These TPD derivatives have also interesting properties such as a quick and easy synthesis, high solubility in common organic solvents and the ability to form excellent films by depositing them through solution process.

## 2. Results and Discussions

### 2.1. Synthesis

**Scheme 1:**
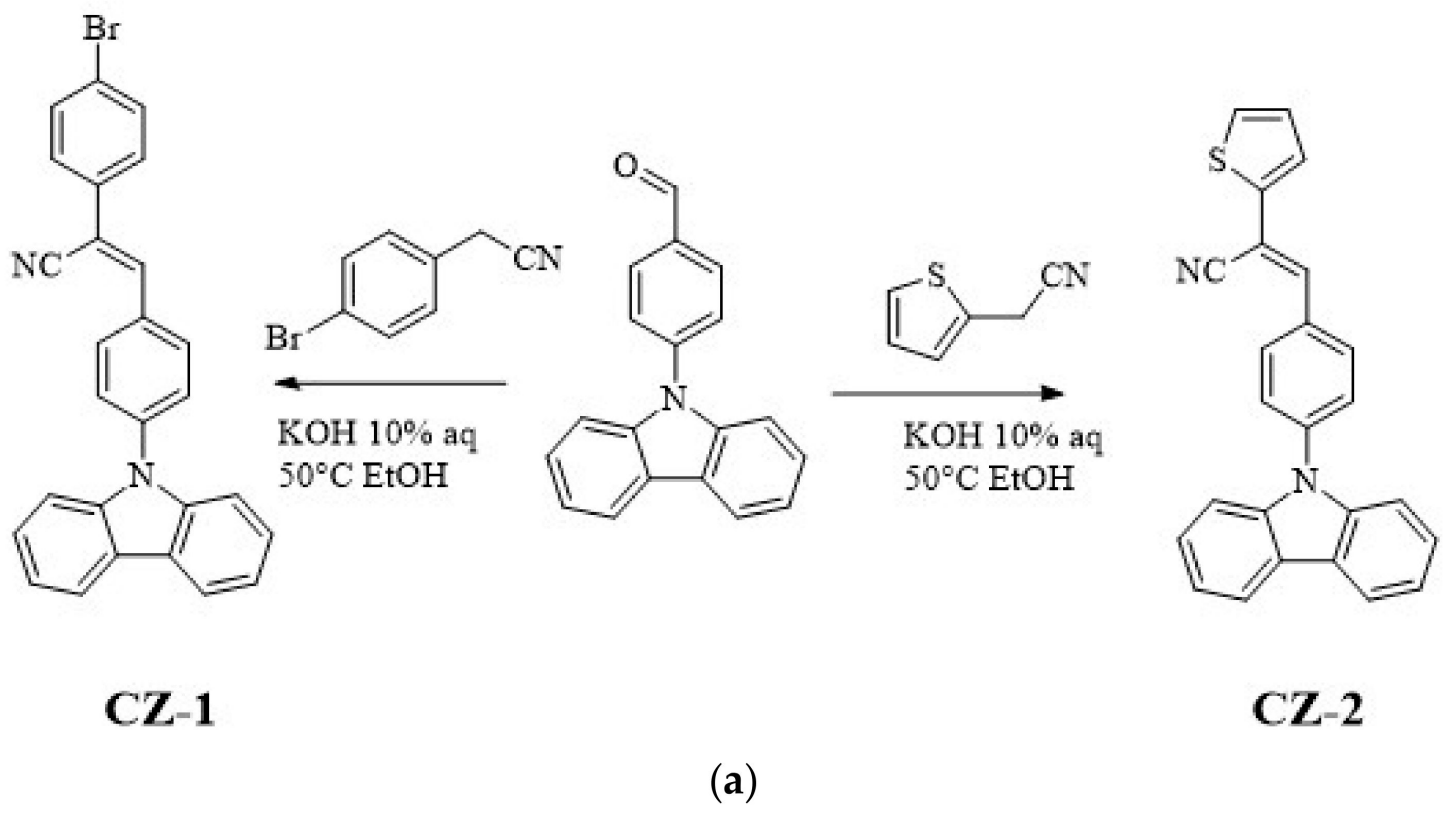

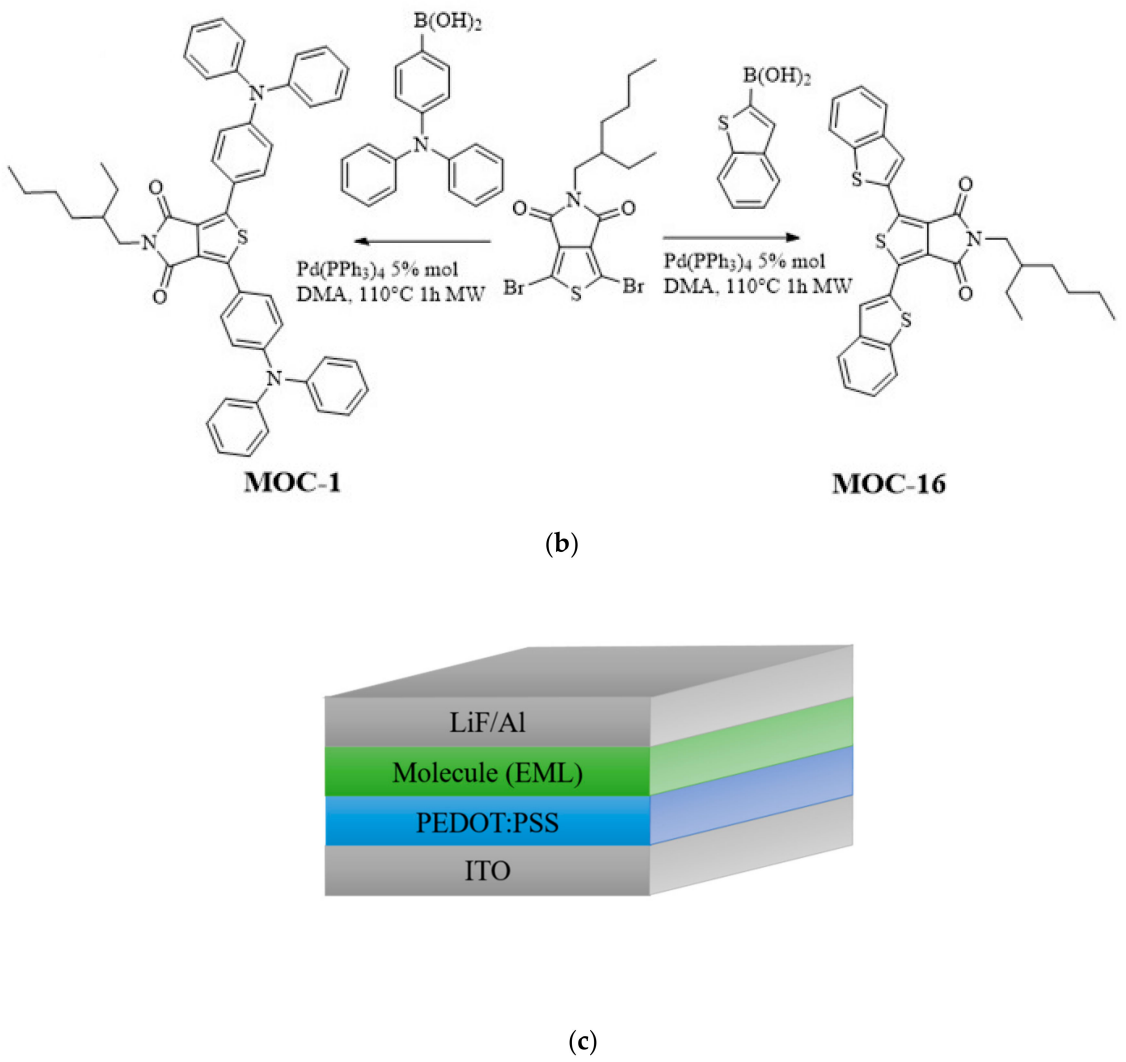
Synthetic route of (**a**) the new low molecular weight molecules derived from carbazole: CZ-2 and CZ-1; (**b**) TPD derivatives: MOC-1 [39] and the new molecule MOC-16; (**c**) used OLEDs architecture.

CZ-2, CZ-1, MOC-1 and MOC-16 molecules, were synthesized according to the modified literature procedure (see Scheme 1) [43,44]; CZ-2 and CZ-1 synthesis was carried out by Knoevenagel reaction between *N*-(4-formylphenyl) carbazole (CZ) and 2-Thiopheneacetonitrile for CZ-2 and 4-Bromophenylacetonitrile for CZ-1. Products were purified by recrystallization; chemical structures were determined by IR, ^1^H, and ^13^C NMR, mass spectrometry. On the other hand the synthesis of MOC-1 and MOC-16 was carried out through the Suzuki reaction between 1,3-Dibromo-5-(2-ethylhexyl)-4*H*-thieno[3,4-*c*]pyrrole-4,6(5*H*)-dione (TPD) and Benzo[*b*]thien-2-ylboronic acid (BT) for MOC-16 and 4-(*N*,*N*-Diphenylamino)-1-phenylboronic acid (TPA) for MOC-1. For the reaction, Pd(PPh_3_)_4_ was used as catalyst in a microwave heating at 110 °C for 1 h with yields of 70%.

### 2.2. Theoretical Calculation

**Figure 1:**
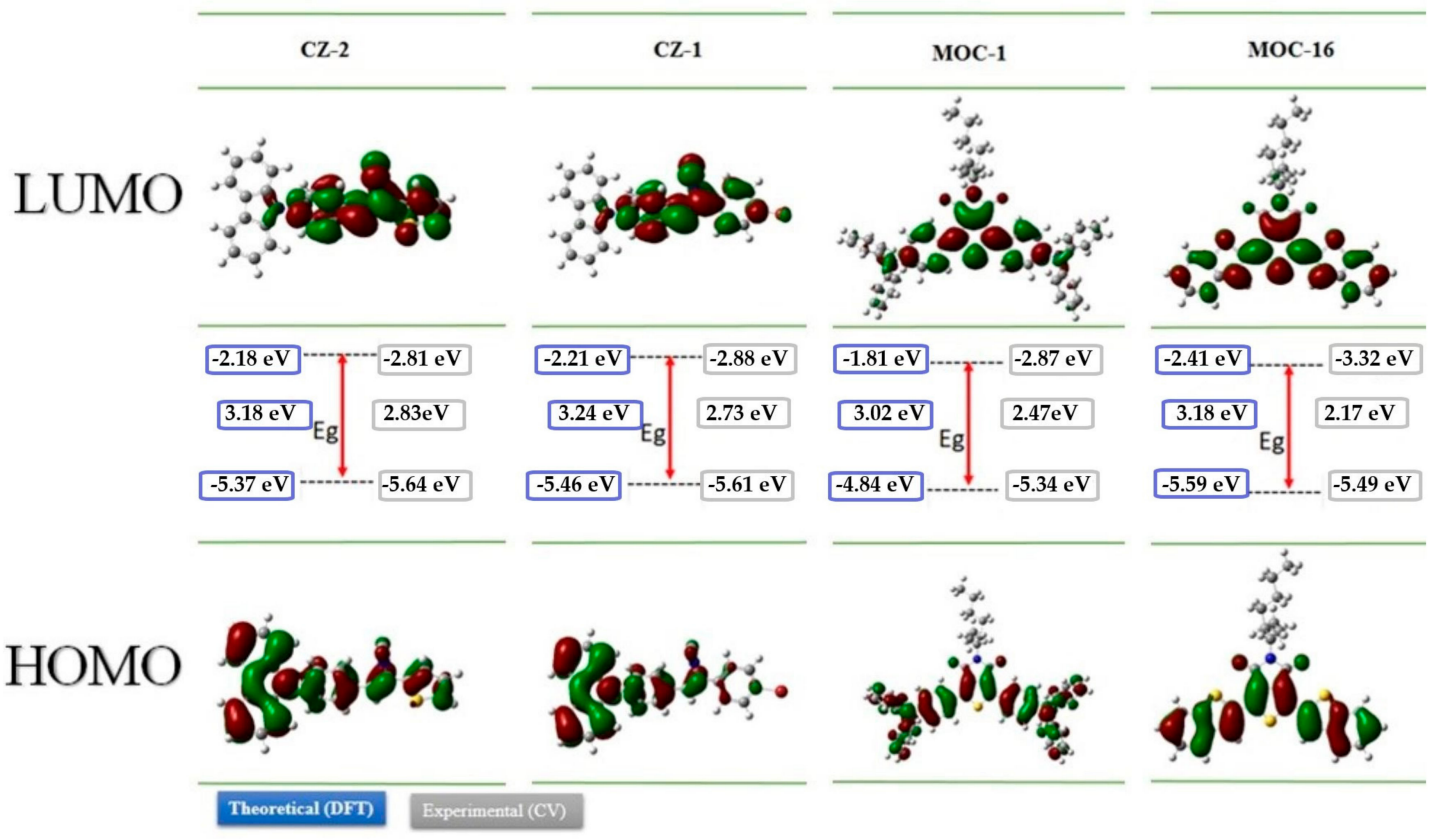
The optimized geometries and electron-state-density distributions and HOMO-LUMO energy levels of CZ-2, CZ-1, MOC-1 and MOC-16 using density functional theory (DFT) at the B3LYP/6-31G* and cyclic voltammetry (CV) measurements.

The geometrical and electronic properties of CZ-2, CZ-1, MOC-1 and MOC-16 were studied by theoretical calculation through the density functional theory (DFT), at the B3LYP/6-31G* level, using the Gaussian 09 package [45]. Figure 1 shows their minimum-energy geometrical conformations and the molecular orbital electron state-density distributions for the highest occupied molecular orbital (HOMO) and lowest unoccupied molecular orbital (LUMO) levels. This Figure 1 also shows the electron density for CZ-2, CZ-1, MOC-1 and MOC-16, it was observed that in their HOMO, this electronic density is mostly localized on carbazole fragment while in their LUMO is localized mainly on the π-conjugated system, indicating that CZ-2 and CZ-1 have dipolar electronic architecture. In contrast, the analysis of the electron density for MOC-1 and MOC-16 shows that both molecules have a DAD quadrupole structure.

In Figure 1 the theoretical and experimental energies values of the HOMO and LUMO levels as well as the band-gap energy (E_g_^cal^) determined by DFT and (E_g_^elec^) measured by electrochemical methods (see next section) are shown. CZ-2, CZ-1, MOC-1 and MOC-16 have E_g_^cal^ values of 3.18, 3.24, 3.02 and 3.18 eV, respectively. These trends of the theoretically calculated energy levels are consistent with the experimental ones, although the values are somewhat different, probably reflecting that the calculations are performed under the gas-phase conditions.

### 2.3. Electrochemical Analysis

**Figure 2:**
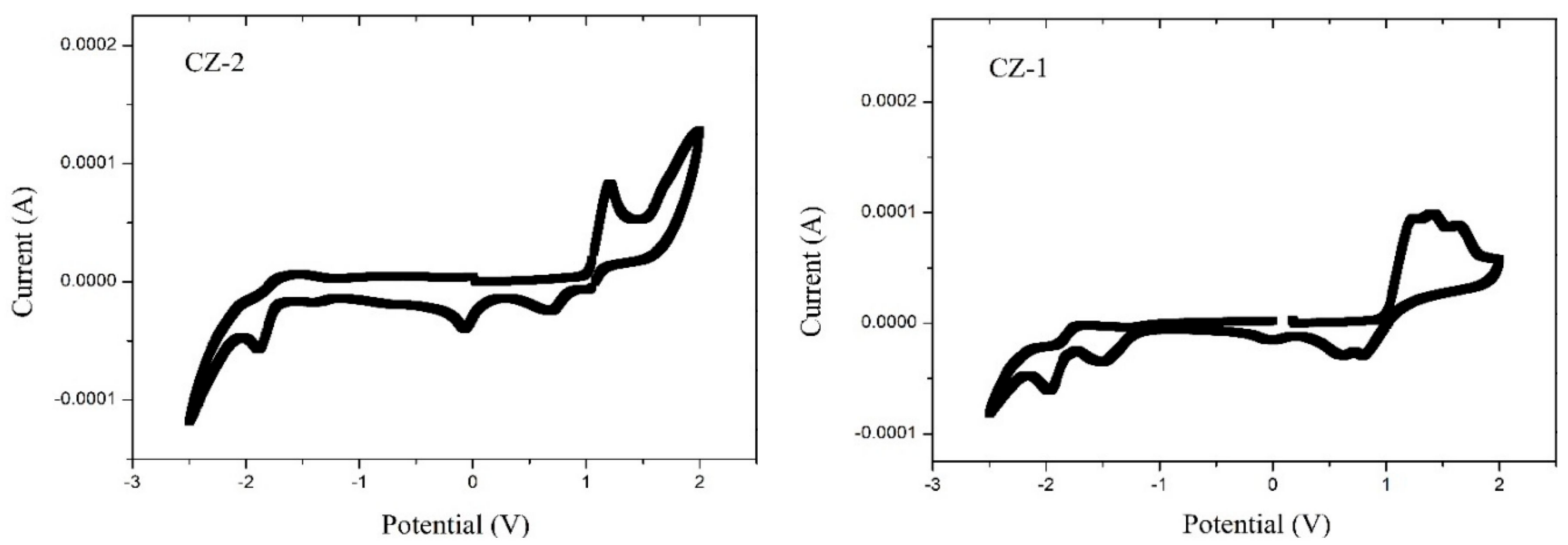

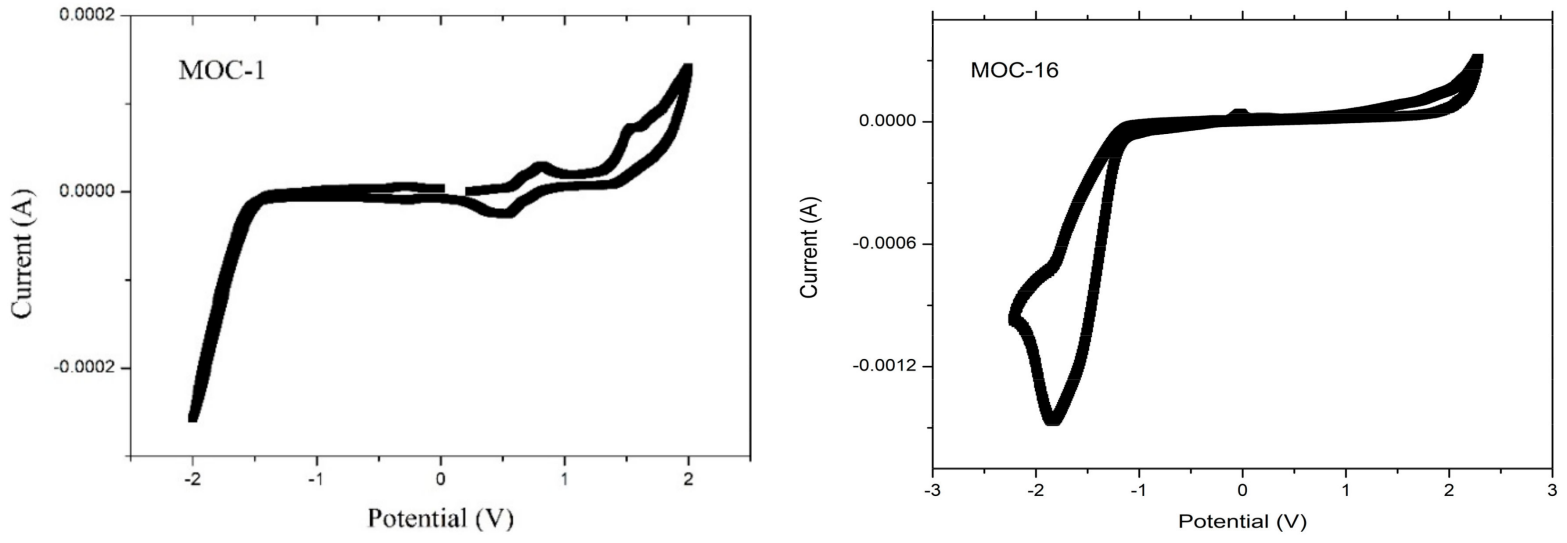
Cyclic voltammograms (50 mV s^−1^) for CZ-2, CZ-1, MOC-1 and MOC-16 compounds.

Recorded CV curves can be shown in Figure 2 for CZ-2, CZ-1, MOC-1 and MOC-16; the oxidation onset potentials ($E_{onset}^{ox}$) referred to ferrocene/ferrocenium (Fc/Fc^+^) are determined to be 1.03, 1.02, 0.68 and 0.87 V, respectively. 

The highest occupied molecular orbital energy level for CZ-2, CZ-1, MOC-1 and MOC-16 was calculated based on the equation E_HOMO_ = −[(E_ox_ + 4.4) + 0.22] eV, where E_ox_ is the recorded onset oxidation potentials of the molecules, 4.4 eV is the energy level of ferrocene/ferrocenium (Fc/Fc^+^) below the vacuum level and 0.22 eV is the correction by the reference used electrode [46]. The HOMO values were −5.65, −5.61, −5.34 and −5.49 for CZ-2, CZ-1, MOC-1 and MOC-16, respectively. The lowest unoccupied molecular orbital levels were determined using the reduction onset potentials $E_{onset}^{red}$ to be −2.81, −2.88, −2.87 and −3.32 for CZ-2, CZ-1, MOC-1 and MOC-16. Theoretical HOMO–LUMO energy values and electrochemical data are summarized in Table 1.

### 2.4. OLED Devices

**Figure 3:**
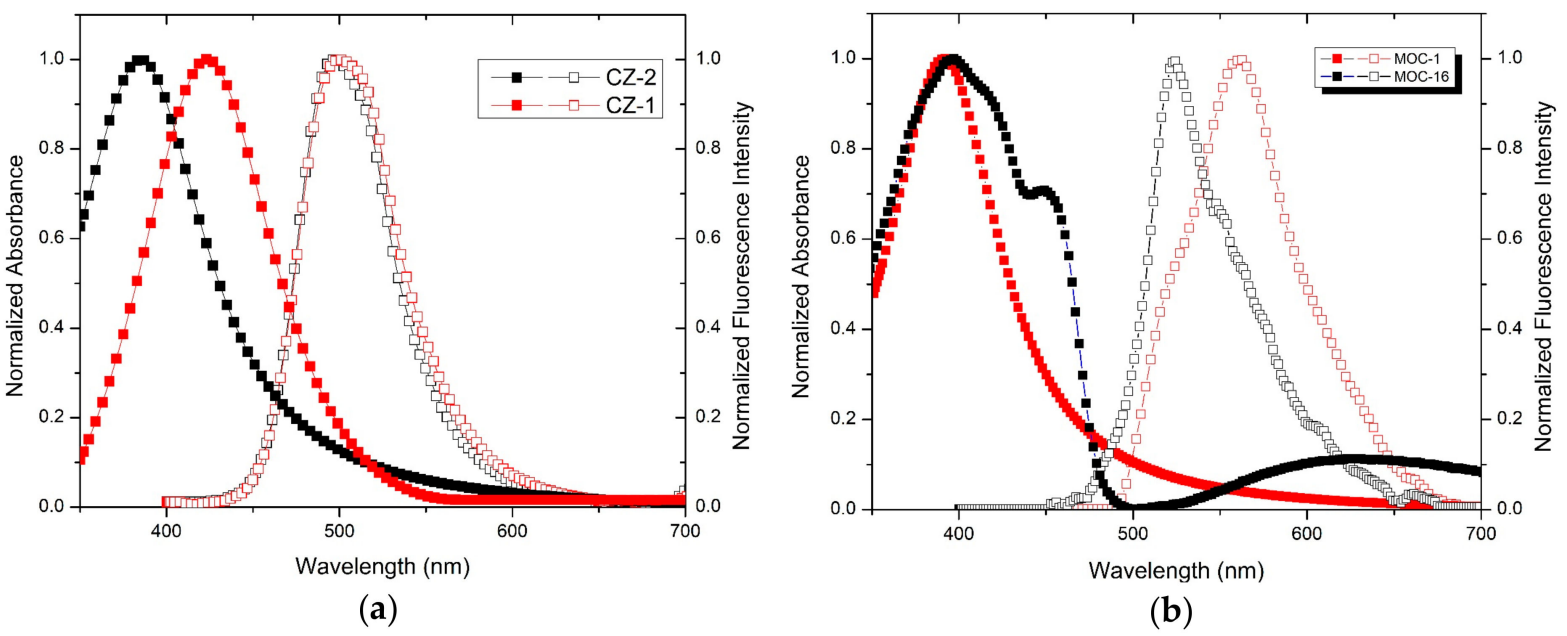
Absorption and fluorescence spectra of (**a**) carbazole derivatives (molecules CZ-2 and CZ-1) and (**b**) TPD derivatives (molecules MOC-1 and MOC-16) in solid state films.

The absorption and fluorescence spectra in solid state films of CZ-2 and CZ-1 are shown in Figure 3a, with a maximum absorption wavelength $\lambda_{max}^{abs}$ at 391 nm for CZ-2 and 430 nm for CZ-1, likewise the spectra of fluorescence show their maximum peaks $\lambda_{max}^{em}\;$ at 498 nm and 499 nm for CZ-2 and CZ-1, respectively. In both cases, contributions of the $n\to \pi^{*}$ transitions are observed by the conjugation of the molecules in their absorption spectra. This is because the n→π* transitions are energetically favored over the transitions π→π* or σ→π*, since less energy is required for their excitation; further, the symmetry of carbazole molecules allows a suitable orbitals orientation for this interaction [47,48]. Absorption and fluorescence spectra of MOC-1 and MOC-16, in solid state films are shown in Figure 3b where MOC-1 has its maximum peak of absorption $\lambda_{max}^{abs}\;$ at 391 nm while MOC-16 at 397 nm, likewise the spectra of fluorescence show their maximum peaks at $\lambda_{max}^{em}$ 523 nm and 561 nm for MOC-1 and MOC-16, respectively.

In both cases, the molecule conjugation is observed at the peaks of the absorption spectra located at 391 nm and 397 nm, the second peak (shoulder) of MOC-16 shifts towards the infrared region. The optical bandgap of CZ-2, CZ-1, MOC-1 and MOC-16 materials were determined from the absorption spectra and values are 2.67, 2.42, 2.68 and 2.57 eV, respectively. These values correlate with those obtained by CV, and are somewhat different from the other calculated values (see Figure 1). 

**Figure 4:**
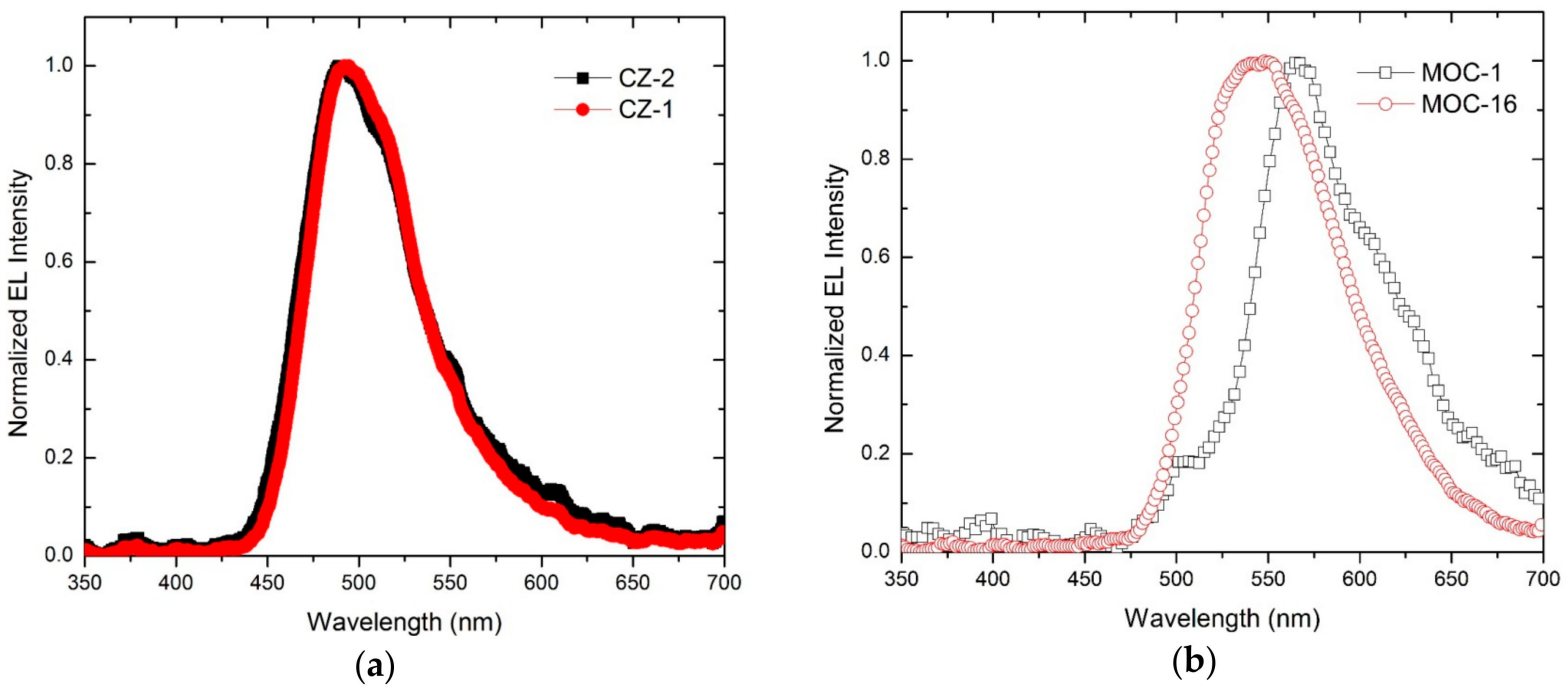
Electroluminiscence spectra of OLEDs based on (**a**) carbazole compounds (CZ-2 and CZ-1) and (**b**) TPD derivatives (MOC-1 and MOC-16) as emissive layers.

Electroluminescence spectra of OLEDs based on CZ-2, CZ-1, MOC-1 and MOC-16 as emissive layers are shown in Figure 4. The OLEDs based on carbazole derivatives have a maximum peak of electroluminescence in 488 nm for CZ-2 and 492 nm for CZ-1 (greenish blue color for both) with Commision Internationale de L’Eclairage (CIE) coordinates (0.17, 0.30) and (0.16, 0.29), with a difference of only 4 nm between their maxima and with a very similar bandwidth, from which the emission color is so similar. Meanwhile, OLEDs with the emissive layer based on TPD derivatives have their maximum peaks at 564 nm and 547 nm for MOC-1 and MOC-16 (with CIE coordinates (0.41, 0.42) and (0.36, 0.60), respectively).

**Figure 5:**
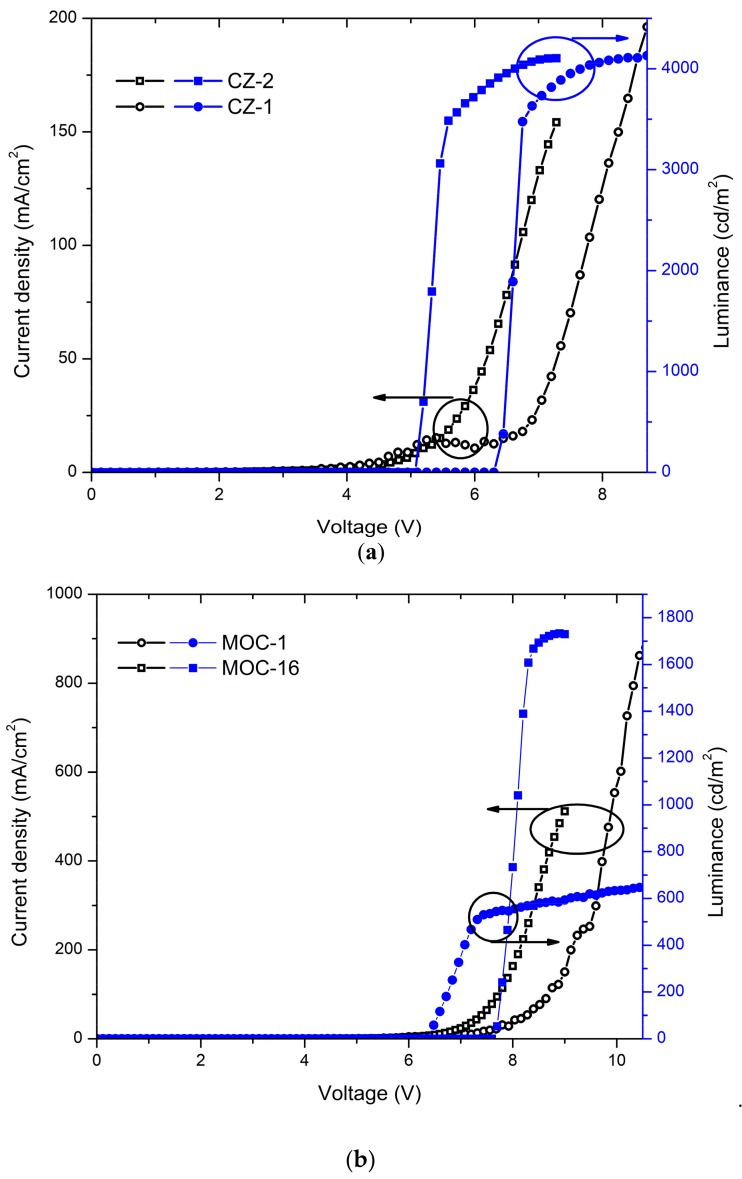

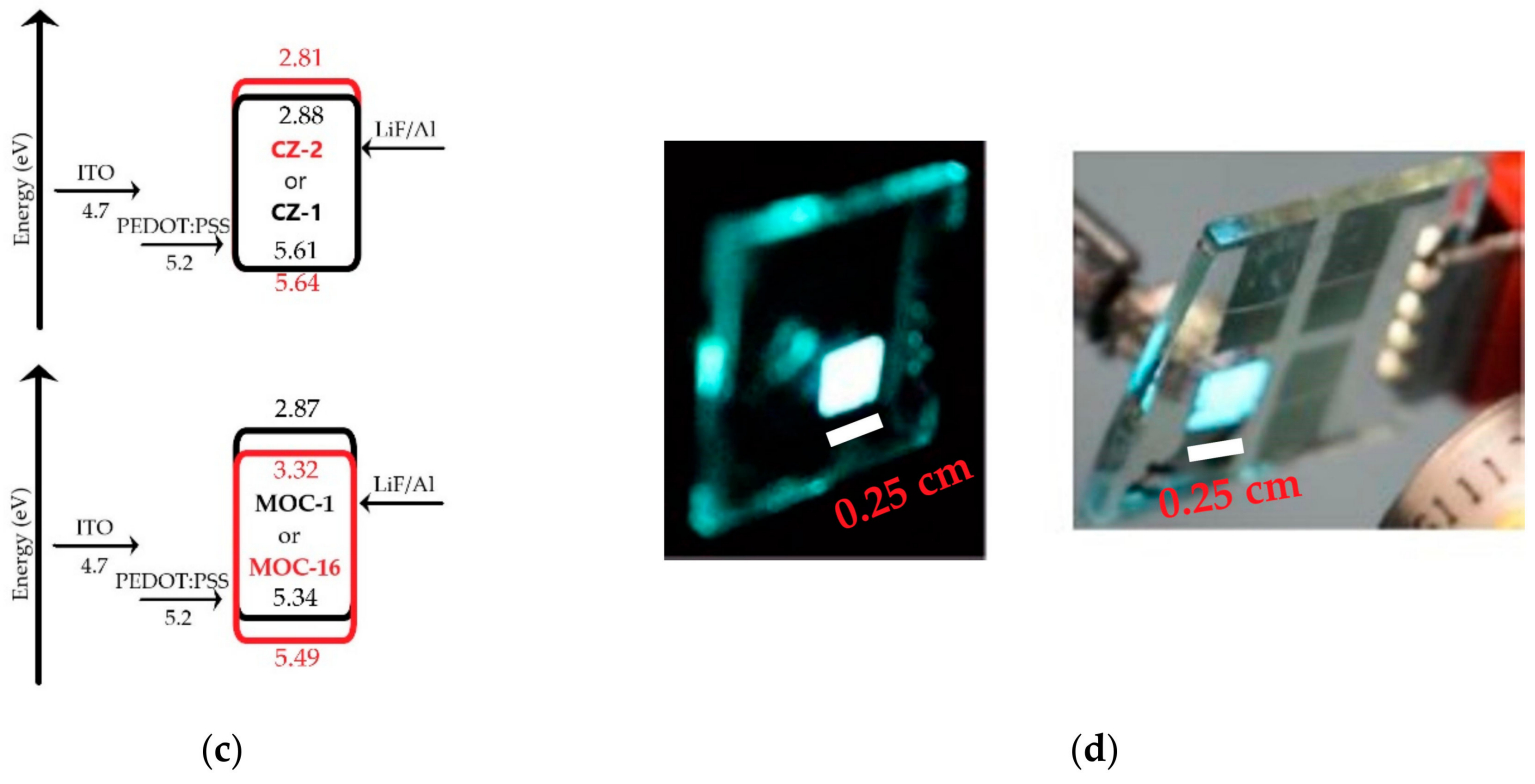
J-V curves and luminances of OLEDs based on (**a**) carbazole derivatives with the configuration ITO/PEDOT:PSS (30–40 nm)/Carbazole derivative (70–80 nm)/LiF (1 nm)/Al (150 nm); here are shown the best values, for deviations/error bars see Figure S8 (SI); (**b**) TPD derivatives with the configuration ITO/PEDOT:PSS (30–40 nm)/TPD derivative (50–60 nm for MOC-1 and 40–50 nm for MOC-16)/LiF (1 nm)/Al (150 nm) (best values); (**c**) schematic energy diagram for OLEDs based on CZ-2, CZ-1, MOC-1 and MOC-16; (**d**) photographs of OLEDs based on CZ-2 (left) and CZ-1 (right) with an area of 2.5 × 2.5 mm^2^.

**Figure 6:**
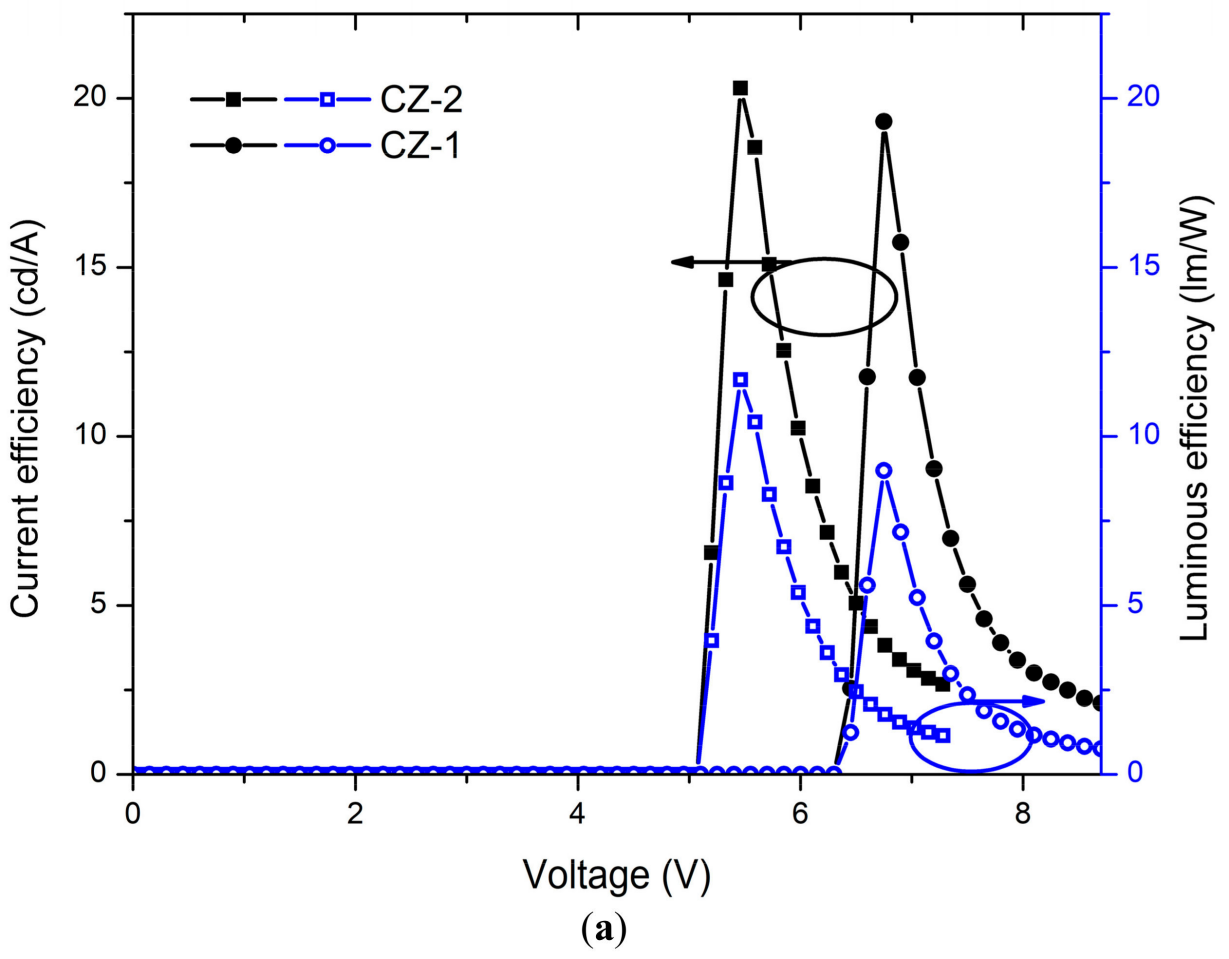

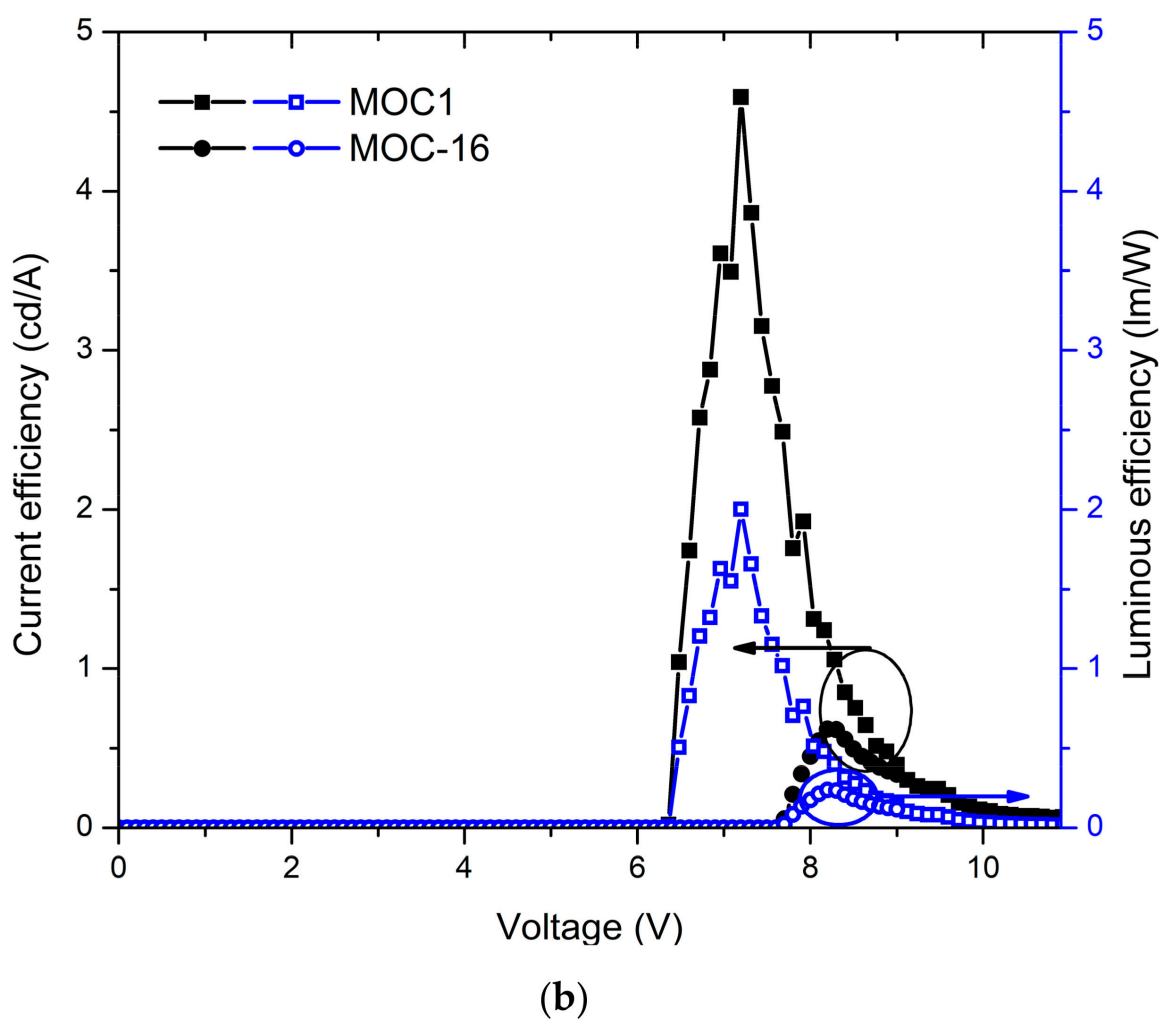
Current and luminous efficiencies of devices based on (**a**) carbazole derivatives (CZ-2 and CZ-1) and (**b**) TPD derivatives (MOC-1 and MOC-16) as emissive layers.

Current-voltage (J-V) curves and luminances (L-V) of OLEDs based on CZ-2 and CZ-1 are shown in Figure 5a while current and luminous efficiencies in Figure 6a. Devices based on CZ-2 (film thickness in the range 70–80 nm) presents a turn on voltage of 5.2 V, as well as a maximum luminance of 4104 cd/m^2^ (at 7.2 V and an current efficiency of 2.6 cd/A), while having a maximum efficiency of 20.2 cd/A at 3062 cd/m^2^. Furthermore, OLEDs based on CZ-1 (film thickness: 70–80 nm) presents a turn on voltage of 6.5 V, a maximum luminance of 4130 cd/m^2^ at 8.7 V and, a maximum current efficiency of 19.3 cd/A at 3476 cd/m^2^. According to the energy diagrams of our devices (Figure 5c), it is observed that electrons are injected by tunneling because LiF creates a potential barrier between the cathode and the emissive layers, increasing the population of electrons, which leads to the increase in the device luminous density.

Also, in Figure 5b the J-V curves and luminances are displayed for OLEDs devices based on MOC-1 and MOC-16, while current and luminous efficiencies are shown in Figure 6b. From the J-V curves of OLEDs based on CZ-2 and CZ-1 is observed that the current density increases in the voltage range 4–9 V, whereas for MOC-1 and MOC-16 it takes place in the range 6.5–10.5 V. Likewise, the maximum current density reached by OLEDs based on carbazole derivatives does not overcome 200 mA/cm^2^, whereas for those based on TPD derivatives the maximum current density is 800 mA/cm^2^. 

Devices with the configuration ITO/PEDOT:PSS (30–40 nm)/MOC-1 (50–60 nm)/LiF (1 nm)/Al (150 nm) had a turn on voltage of 6.2 V, and a maximum luminance of 651 cd/m^2^ (at 10.6 V) at a current efficiency of 0.07 cd/A while in their point of maximum current efficiency (4.5 cd/A) had a luminance of 467 cd/m^2^. Moreover, devices with the configuration ITO/PEDOT:PSS (30–40 nm)/MOC-16 (40–50 nm)/LiF (1 nm)/Al (150 nm) showed a turn on voltage of 7.7 V and, a maximum luminance of 1792 cd/m^2^ (at 7.9 V) at a current efficiency of 0.34 cd/A, while the maximum current efficiency is 0.61 cd/A (at 1388 cd/m^2^). 

**Figure 7:**
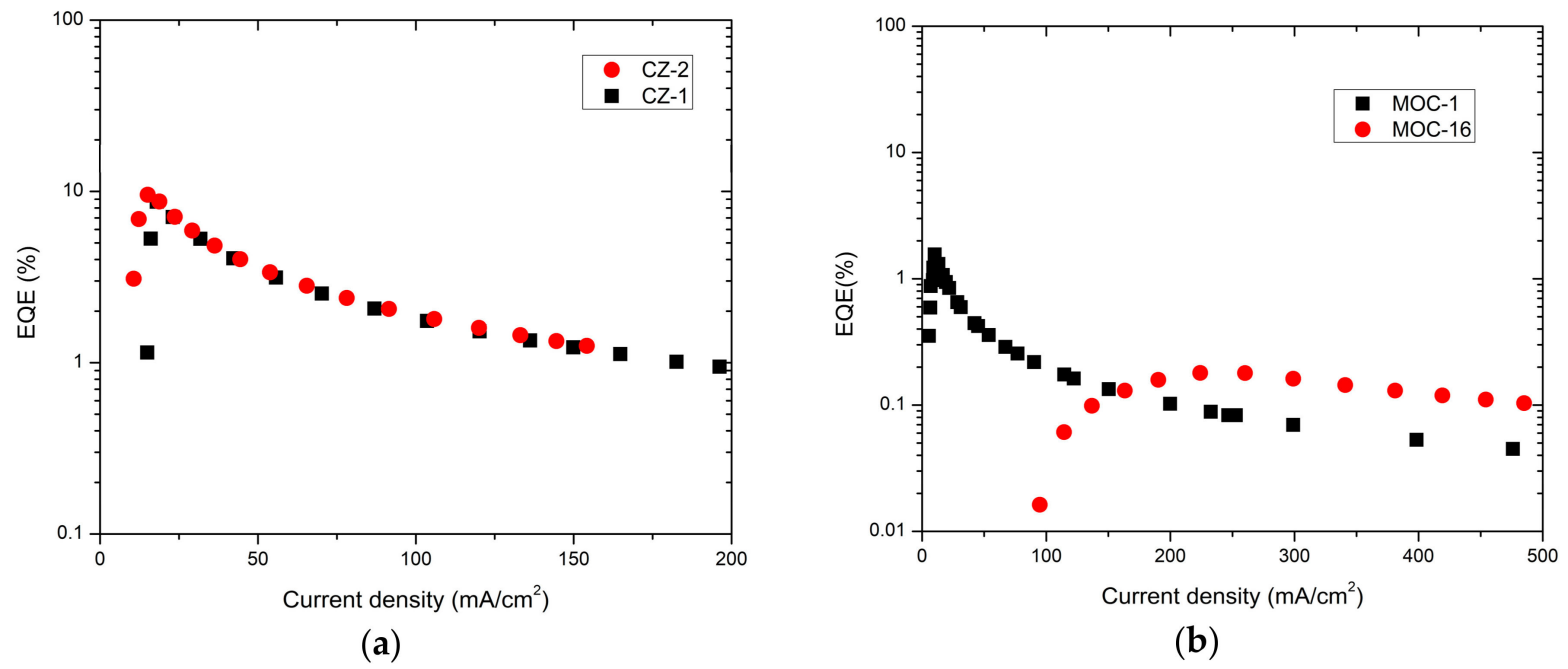
EQE of (**a**) devices based on carbazole derivatives (CZ-2 and CZ-1) and (**b**) TPD derivatives (MOC-1 and MOC-16).

For OLEDs based on carbazole derivatives, cautiously, a very impressive maximum EQE of 9.5% and 8.6% was determined (Figure 7a) for CZ-2 and CZ-1, respectively; in both cases around 20 mA/cm^2^. For OLEDs based on TPD derivatives it was estimated a maximum EQE of 1.5% and 0.1% for MOC-1 and MOC-16 respectively (Figure 7b). These EQE values for carbazole derivatives need to be compared with other previous reported values for different compounds (see Table 2). For instance, devices based on G3MP (A3 and B3) [21] have a maximum EQE of 12.8% and 10.3%, respectively, both with their EML as host/guest type. M2 [49] and B [50] devices with only the carbazole derivative as emitter (like our devices) have a maximum EQE 3.0% and 3.6% respectively (with EL emission around the deep blue). OLEDs with better performance have maximum EQE of 20.1% and 14.4% for DCZ-TTR [34] and CZ-TTR [34] respectively, using as EML a host/guest type; they showed similar EL emission, however, such devices have low operation voltage and low current densities values. 

Table 2 shows the main OLEDs parameters for our reported devices based on carbazole and TPD moieties, as EML, as well as some examples/comparisons from previous reports of OLEDs devices with some similarities such as: emission bands, manufacturing methods, derivatives of the same material, architectures, etc.

**Figure 8:**
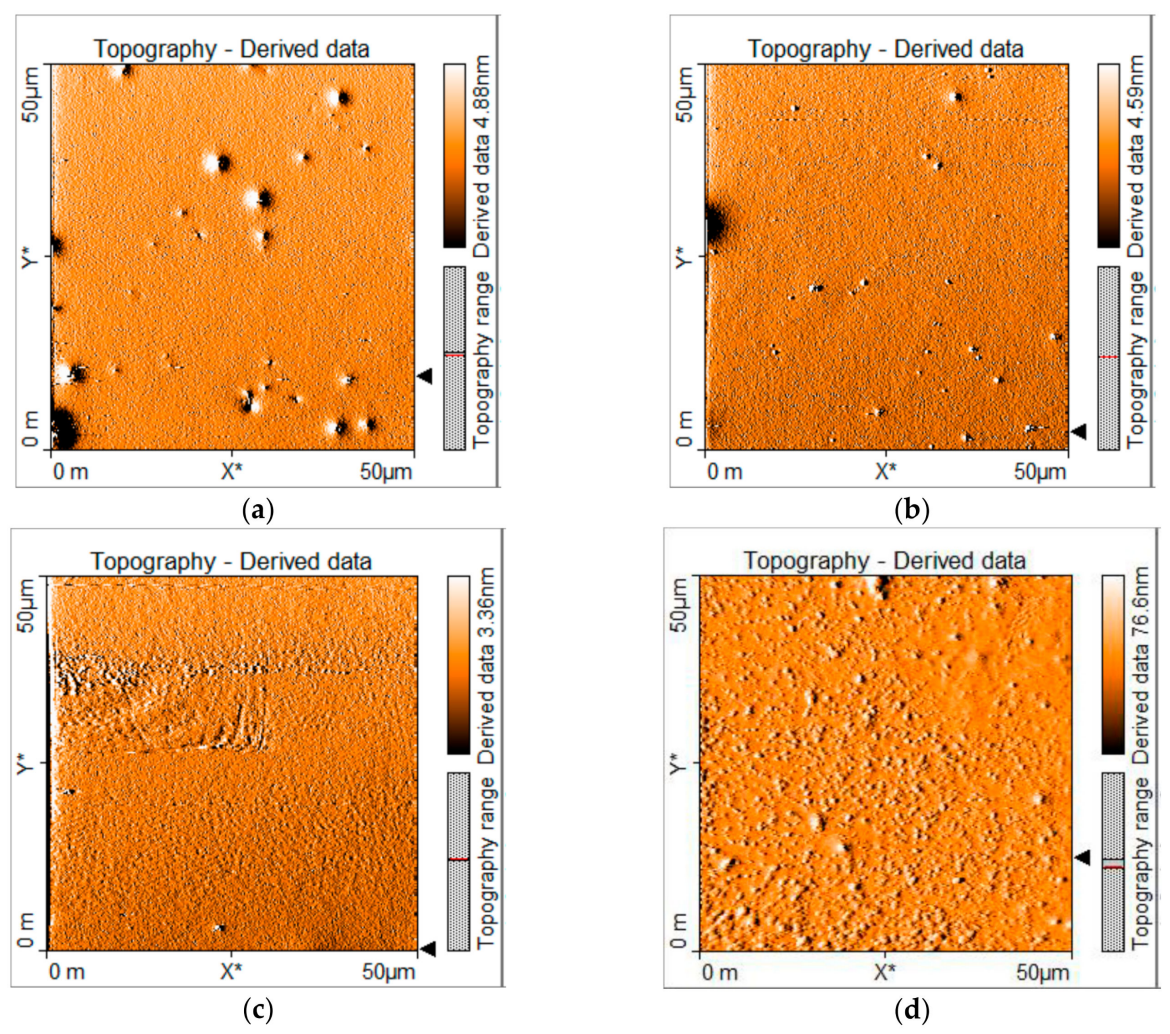
AFM images of deposited layers by spin coating of (**a**) CZ-2, (**b**) CZ-1, (**c**) MOC-1 and (**d**) MOC-16 molecules. ITO/PEDOT:PSS/EML architecture, as in our OLED devices, was used for these AFM measurements.

As it is clear, OLEDs based on carbazole derivatives as emissive layers have larger luminance and efficiency than those based on TPD derivatives. One reason could be because devices based on the latter compounds showed some agglomerations and/or film deformities as can be seen from AFM images (see Figure 8), with roughness of 1.8 nm in the case of MOC-1 and in the case of MOC-16 13 nm, which may provoke the high current densities (400 to 850 mA/cm^2^) (see Figure 5b), and therefore, the low efficiencies (4.5 cd/A and 0.61 cd/A, respectively). On the other hand, OLEDs based on carbazole derivatives have a low roughness in the film (2 nm) like MOC-1, low current densities (<200 mA/cm^2^) (see Figure 5a), very acceptable luminance values (>4000 mA/cm^2^) and good current efficiencies (20.2 cd/A and 19.3 cd/A, for CZ-2 y CZ-1, respectively). Also, in solid state thin films, CZ-1 shows a reasonable high value of its fluorescence quantum yield (PLQY): 51.9% (see Figure S5 in SI).

From the chemical structure point of view, for these studied small molecules, some factors that may influence the described behavior (better electroluminescence properties and efficiency for CZ-2 and CZ-1 compounds than for MOC-1 and MOC-16) are: dipolar structure for the case of CZ-2 and CZ-1 (carbazole derivatives) and quadrupolar for MOC-1 and MOC-16 as well as the carbazole fragment could impact a different packing, thus, enhancing better crystallinity (and charge carrier transport) than with the diphenyl part of the quadrupolar compounds. Finally, according to the energy diagram showed in Figure 5c), there exist a better energy level alignment of CZ-2 and CZ-1 with the HOMO-LUMO levels of the other compounds than for the case of MOC-1 and MOC-16.

When comparing the reached results for our OLEDs devices based on CZ-2 and CZ-1 as EML with those reported in the literature, it can be observed that these measured current efficiencies of 19.3 cd/A and 20.2 cd/A, respectively, are in general similar (or even larger) than those for other OLEDs using also compounds derived from carbazole as emitter material, see Table 2. For instance: M2 [49], B [50] and CP3 [32] (by spin coating as well as by evaporation), have current efficiencies of 1.53 cd/A (at 4543 cd/m^2^), 1.8 cd/A (and maximum luminance up to 2267 cd/m^2^) and 2.53 cd/A (with maximum luminance up to 8235 cd/m^2^), respectively. This trend was because these OLEDs had larger current densities compared to those for our devices. Also, these previous reported OLEDs showed similar turn on voltages. Furthermore, under more robust architectures than those for our devices, larger luminescent values are reached, as in the case of the OLEDs based on Blue-1 [51] (host approach) that has two buffer layers and excellent coupling of energy levels; what favors their emission and thus reaching 19,283 cd/m^2^. For Dev. 2 [52], authors reported an EQE_max_ of 4.71% for a device with the architecture ITO/PEDOT:PSS/2/Ca/Al. On the other hand, for an OLED under the configuration ITO/BITPI/LiF/Al [53] it was reported an EQE_max_ of 2.98%, however they reached more of 7% (EQE_max_) for a more robust architecture device (multilayer) based on the same emitter BITPI. In both mentioned articles, device emission is located into the deep blue region (452 nm) where; in general, efficiency is lower in comparison with the wavelength range of our devices (greenish blue). It is because blue emissive materials possess a larger band gap and the charge injection is more difficult [4]. However, one of the most efficient devices are those based on TCBzC (as EML) [36], DCZ-TTR (as host) [34] and CZ-TTR (as host) [34], which presents a very high current efficiencies (31.6, 59.6 and 32.5 cd/A, respectively), low turn on voltages (2.5, 3.2 and 3.1 V, respectively) very acceptable luminance values (9226, ~5000 and ~1000 cd/m^2^, respectively) as well as EQE values up to 20.1% and 14.4% for DCZ-TTR and CZ-TTR, respectively. Likewise, OLEDs devices were recently reported based on carbazole derivatives “Dev. III no. 2” [37] and TPE-DFCz [38], with emission wavelengths (492 and 500 nm, respectively) similar to those for our OLEDs based on CZ-2 and CZ-1, and having maximum luminance within the range 4390–3200 cd/m^2^; however, with lower current efficiencies: 1 and 1.16 cd/A versus 20.2 and 19.3 cd/A, respectively. 

It should be noted that devices with the highest efficiency are usually those that use carbazole derivatives in their active films under the host/guest approach, for instance: OLEDs based on Blue-1 [51] and G3MP (A3 and B3 devices) [21], whose luminances are 19,283 cd/m^2^ and 9823 cd/m^2^, respectively, with current efficiencies from 10.8 cd/A until 28.2 cd/A, i.e., similar to those values for our OLEDs. Also, devices based on G3MP(A3) [21] and G3MP(B3) [21] as emissive layer, presented EQE values of 12.8% and 10.3%, respectively. However, we are using a simple EML (deposited by spin coating) not a host/guest type emission layer. Here, our OLED devices could be considered as double-layer diodes (PEDOT:PSS layer and the ultra-thin LiF film act as hole injector layer/electron blocking layer and as an electron injector layer, respectively).

Regarding our determined EQE_max_ values, it is true that these high EQE_max_ (8.6–9.5%) are rather impressive values for our fabricated fluorescent OLEDs based on CZ molecules. According to Adachi et al. [54]: “EQE (η_EQE_) is the product of the internal quantum efficiency (IQE) and light out-coupling factor (η_out_), η_EQE_ = IQE × η_out_; η_out_ is typically 20–30%. IQE is obtained from IQE = β × γ × Φ_PL_, where β is the exciton generation factor resulting in photons, γ is the carrier balance ratio of holes and electrons, and Φ_PL_ is the photoluminescence (PL) quantum yield (PLQY). Because only singlet excitons generate photons, the resulting β for fluorescent OLEDs is limited to 25%. Even under conditions where γ and Φ_PL_ are 100%, the theoretical maximum EQE of the resulting OLED is only 5–7.5%”. Thus, additional work is necessary to elucidate and clarify acceptable reasons of these cautious determined high EQE_max_ values in this current article.

## 3. Materials and Methods

### 3.1. Materials

Potassium hydroxide was purchased from J.T. Baker: Phillipsburg, New Jersey; 4-Bromophenylacetonitrile was acquired from Alfa Aesar: Haverhill, Massachusetts, 2-Thiopheneacetonitrile, 1,3-Dibromo-5-(2-ethylhexyl)-4*H*-thieno[3,4-*c*]pyrrole-4,6(5*H*)-dione, tetrakis(triphenylphosphine)palladium (Pd(PPh_3_)_4_), *N*-(4-formylphenyl)carbazole, Benzo[*b*]thien-2-ylboronic acid, 4-(*N*,*N*-Diphenylamino)-1-phenylboronic acid, *N*,*N*-dimethylacetamide and THF were purchased from Aldrich: Toluca, Estado de Mexico. The others reagents and solvents were purchased from Karal: León, Guanajuato. All reagents and chemicals were used without further purification unless stated otherwise.

### 3.2. Synthesis of Materials

#### 3.2.1. General Method for Knoevenagel Reaction

Ten mmol of the acetonitrile derivate and 10 mmol of *N*-(4-formylphenyl) carbazole are placed in a 50-mL flask, 25 mL of ethanol are added. Then the mixture is stirred for 5 min, after that, 3 drops of an aqueous solution 10% of potassium hydroxide is added to the mixture, the reaction is stirred and heat up to 50 °C, and was monitored by thin layer chromatography until was completed, then the mixture is cool down to 0 °C. The formation of a crystalline precipitate is observed, it is filtered and washed using cold EtOH. The solid is recovered and purified by recrystallization process using as solvent a mixture of acetone/EtOH 40:60.

*(E)-3-(4-(9H-carbazol-9-yl)phenyl)-2-(thiophen-2-yl)acrylonitrile* (CZ-2): Yellow solid, Yield: 90%, m.p. 159–161 °C. FTIR (ATR, cm^−1^) 2214 (C≡N), 1487 (C−S). ^1^H NMR (500 MHz, THF-*d*_6_) *δ *(ppm): 8.07–8.21 (4H, m), 7.62–7.77 (3H, m), 7.32–7.52 (6H, m), 7.16–7.26 (2H, m), 7.04–7.13 (1H, m); ^13^C NMR (125 MHz, THF-*d*_6_) *δ *(ppm): 140.2, 139.4, 139.2, 132.4, 130.6, 127.9, 127.1, 126.8, 126.7, 125.9, 123.8, 120.2, 120.1, 116.4, 109.6, 106.1; HRMS–TOF (ESI, *m*/*z*): [M + H]^+^ Found: 377.11064 for C_25_H_16_N_2_S; [M + H]^+^ Calcd: 377.11069; Error 0.1987 ppm.

*(Z)-3-(4-(9H-carbazol-9-yl)phenyl)-2-(4-bromophenyl)acrylonitrile* (CZ-1): Yellow solid, Yield: 98%, m.p. 224–225 °C. FTIR (ATR, cm^−1^) 2212 (C≡N), 1230 (C−N). ^1^H NMR (500 MHz, CDCl_3_) *δ *(ppm): 8.12–8.15 (4H, m), 7.71 (2H, d, *J* = 8.6 Hz), 7.57–7.62 (5H, m), 7.50 (2H, d, *J* = 8.3 Hz), 7.41–7.45 (2H, m), 7.30–7.34 (2H, m); ^13^C NMR (125 MHz, CDCl_3_) *δ *(ppm): 141.2, 140.2, 139.8, 133.2, 132.3, 132.0, 130.9, 127.5, 126.9, 126.2, 123.7, 123.6, 120.5, 120.4, 117.5, 110.9, 109.8; HRMS-TOF (ESI, *m*/*z*): [M + H]^+^ Found: 449.06469 for C_27_H_17_BrN_2_; [M + H]^+^ Calcd: 449.06478; Error 0.1483 ppm. 

#### 3.2.2. Suzuki Reaction

In a flask of 20 ml, the diboronic acid and the 1,3-Dibromo-5-(2-ethylhexyl)-4*H*-thieno[3,4-*c*]pyrrole-4,6(5*H*)-dione are placed, subsequently 3 molar equivalents of potassium pivalate (PIVOK) are added and finally 0.05 mol % of catalyst tetrakistriphenylphosphine Pd(PPh_3_)_4_ was added. The mixture was placed under nitrogen atmosphere, then 5 mL of dimethylacetamide (DMA) is added; the mixture was stirred for 2 min and then heated up in the microwave at 110 °C with a power of 100 W. After 1 h, the reaction was cooled down to 50 °C and 50 mL of cold EtOH are added, a solid precipitate is observed, which is filtered and washed first with 50 mL of EtOH and then with 50 mL of hexane. The solid was recovered and recrystallized using a mixture of ketone/EtOH 40:60.

1,3-bis(4-(diphenylamino)phenyl)-5-(2-ethylhexyl)-4*H*-thieno[3,4-*c*]pyrrole-4,6(5*H*)-dione (MOC-1): see Reference [39].

1,3-bis(benzo[*b*]thiophen-2-yl)-5-(2-ethylhexyl)-4*H*-thieno[3,4-*c*]pyrrole-4,6(5*H*)-dione (MOC-16): Yellow solid, Yield: 68 %, m.p. 167–169 °C. FTIR (ATR, cm^−1^) 2957, 2928, 2870, 2858 (C-H), 1698 (C=O). ^1^H NMR (500 MHz, THF-*d*_6_) *δ* (ppm): 8.50 (2H, s), 7.83–7.91 (4H, m), 7.33–7.40 (4H, m), 3.52–3.59 (2H, d, *J* = 7.5 Hz), 1.70–1.82 (1H, m), 1.22–1.41 (8H, m), 0.84–0.92 (6H, m); ^13^C NMR (125 MHz, THF-*d*_6_) *δ* (ppm): 140.6, 140.1, 136.4, 131.7, 130.9, 127.0, 126.1, 125.1, 124.6, 122.1, 41.9, 38.5, 30.7, 28.7, 23.9, 22.9, 13.5, 9.9, 162.2 (C=O).; HRMS-TOF (ESI, *m*/*z*): [M + 2H]^+^ Found: 531.40596 for C_30_H_27_NO_2_S_3_; [M + 2H]^++^ Calcd: 531.40603; Error 0.1574 ppm.

### 3.3. Electrochemical Analysis

Electrochemical properties were evaluated by cyclic voltammetry (CV). All reported potentials were calibrated against the ferrocene/ferrocenium (Fc/Fc^+^) couple, which was used as the internal standard.

### 3.4. Fabrication of the OLED Devices

OLED devices (see Scheme 1c) were fabricated on glass substrates covered with indium-tin oxide ITO, which were cleaned with soap, ethanol and acetone in a sonicator. Subsequently, plasma-oxygen treatment was given for 8 min. Poly(2,3-dihydrothieno-1,4-dioxin)-poly(styrenesulfonate) (PEDOT:PSS, CLEVIOS P VP Al 4083) was deposited by spin-coating on the substrate, with a thickness of 30–40 nm, a thermal annealing at 120 °C was provided for 15 min to remove the residual solvent. Within a controlled nitrogen atmosphere, emissive layers (EML) were deposited by spin-coating at different speed to reach the best performance for each EML. By the spin-coating technique, film thickness reproducibility could be around ±8 nm, it is because it is necessary to take care of different experimental details such as speed (RPM), different solvents, concentration (viscosity), initial solution deposition, solution amount, ramp time, plateau time, temperature control, different substrates and their cleaning, and previous treatment, etc. CZ-2, MOC-1 and MOC-16 were dissolved in chlorobenzene at a concentration of 36 mg/mL, while CZ-1, at the same concentration, in a mixture of chlorobenzene and chloroform (20 wt %) in order to change the polarity of the used solution, it was because CZ-1 is not easily dissolved in pure chlorobenzene in comparison to CZ-2. It was used around 10–15 µL of solution for the EML deposition for each substrate. The speed for CZ-1 and CZ-2 thin film formation had a variation from 1000 to 2500 rpm, depending on the desired thickness; for instance, it was used 2000 rpm to reach 70–80 nm for CZ-1 and 1500 rpm to obtain 70–80 nm of thickness for CZ-2. The emissive layers received thermal annealing at 80 °C for 20 min. Evaporation of 1 nm thickness of LiF was at the rate of 0.01–0.03 Å/s and 150 nm of Al were evaporated at a rate of 0.05 Å/s for the first 30 nm, 0.08 Å/s for the next 70 nm and at 1–1.5 Å/s for the last 50 nm. Morphological and film thickness (for the organic layers) measurements were performed by atomic force microscopy (AFM) (Nanosurf, easyscan2: Liestal, Switzerland) operating in contact mode (when measuring film thickness through AFM, another (additional to the ±8 nm when deposited by spin cast) error about ±2 nm could be introduced). Initially, OLEDs with different EML thickness were fabricated (usually in series of 3–4 devices each one), see Figure S7 (SI). Based on these experiments, the EML thickness was chosen according to the measured current density and luminance values. For realizing of reproducibility/errors, other series of 3–4 devices for each EML type were again fabricated and tested; in general the OLEDs performance followed a similar trend (see Figure S8 (SI); where it can be seen the average values and errors bars/deviation of the J-V-L plots for CZ1 and CZ-2 EMLs).

### 3.5. OLEDs Characterization

The current density versus voltage (J-V) plots and luminous efficiency versus voltage (L-V) curves were measured simultaneously using a power supply (Keithley 2400, Cleveland, Ohio) with an in-house-designed and calibrated detection system. The J-V curve was recorded by direct processing of data acquired from the used Keithley 2400 apparatus. Luminous density was estimated through the voltage delivered by a photodiode located at fixed distance from the OLED. Photodiode calibration was performed by using the luminance of commercial LEDs, at different wavelengths and considering all geometrical parameters involved in the detection system. Signal was previously quantified by a highly sensitive lux meter and correlated with the photodiode voltage response. All data acquisition routines were automated by using LabVIEW software specially designed for this purpose [55]. External quantum efficiency (EQE) was determined from the J-V, L-V plots and the electroluminescence (EL) spectrum, through the elementary approach reported by Shukla M. et al. [56] and S. Chen [57]. OLEDs emission was characterized under room conditions.

## 4. Conclusions

These new, low molecular weight carbazole derivatives (CZ-2 and CZ-1) and the TPD derivatives (the already reported MOC-1 and the new MOC-16) are of an easy, economical and fast synthesis. They have good solubility and show good film formation (except MOC-16) by solution process. Efficient OLEDs (>20 cd/A) with these carbazole derivatives (CZ-2 and CZ-1 with dipolar structure) were fabricated with the emissive layers deposited by the spin-coating method; they showed luminances greater than 4000 cd/m^2^ and electroluminescence in the greenish-blue region. In comparison with previous similar OLED devices (either under the EML or the host/guest approach), our results for OLEDs based on carbazole derivatives had very acceptable current efficiencies due to a low generated current density value (<200 mA/cm^2^). On the other hand, devices manufactured with TPD derivatives (with quadrupolar structure) showed luminances up to 1729 cd/m^2^ in the case of MOC-16. Nevertheless, current efficiencies are just 4.5 cd/A for the case of MOC-1.

## Figures and Tables

**Table 1:** Theoretical and experimental HOMO-LUMO energy levels (eV) of CZ-2, CZ-1, MOC-1 and MOC-16 using DFT at the B3LYP/6-31G* level and cyclic voltammetry.

	Calculated			Experimental		
	HOMO (eV)	LUMO (eV)	Eg^cal ^(eV)	HOMO (eV)	LUMO (eV)	Eg^exp ^(eV)
CZ-2	−5.37	−2.18	3.18	−5.64	−2.81	2.83
CZ-1	−5.46	−2.21	3.24	−5.61	−2.88	2.73
MOC-1	−4.84	−1.81	3.02	−5.34	−2.87	2.47
MOC-16	−5.59	−2.41	3.18	−5.49	−3.32	2.17

**Table 2:** Device performance of the four kinds of OLEDs based on carbazole and TPD derivatives as EML ^†^. Several other devices from the literature are also mentioned.

EML	$V_{on}\left(V\right)$	$L_{max}\left(\frac{cd}{m^{2}}\right)$	$V_{L_{max}}\left(V\right)$	$\eta_{L_{max}}\left(\frac{cd}{A}\right)$	$\eta_{max}\left(\frac{cd}{A}\right)$	$L_{\eta_{max}}\left(\frac{cd}{m^{2}}\right)$	EQE_max _(%)	$\lambda_{EL}\;\left(nm\right)$	**Reference**
CZ-2 ^d^	5.2	4104	7.2	2.6	20.2	3062	9.5	488	**This work**
CZ-1 ^d^	6.5	4130	8.7	2.1	19.3	3476	8.6	492	**”**
MOC-1 ^d^	6.2	651	10.6	0.07	4.5	467	1.5	564	**”**
MOC-16 ^d^	7.7	1729	7.9	0.34	0.61	1388	0.1	547	**”**
CP3 ^(a),(c),m^	3	8235	8.8	---	2.53	---	---	474	[32]
CP3 *^,(a),(c),m^	2.7	24,442	9	---	6.9	---	---	501	[32]
TCBzC ^(a),m^	2.5	9226	---	---	31.6	---	---	534	[36]
Blue-1 ^(b),m^	3.6	19,283	---	---	10.8	---	---	~460	[51]
G3MP(A3) ^(b),m^	5.3	9823	---	---	28.2	---	12.8	~480	[21]
G3MP(B3) ^(b),m^	4.5	2227	---	---	18.2	---	10.3	~480	[21]
M2 ^(a),m^	3.4	4543	---	---	1.53	---	3.0	428	[49]
B ^(^^a),m^	3.8	2267	---	---	1.8	---	3.6	436	[50]
Dev. III no. 2 ^(^^a),(^^c),d^	4.6	4390	---	---	1.0	---	0.4	492	[37]
TPE-DFCz ^(a),(c),^^d^	5.4	3200	---	---	1.16	---	0.4	500	[38]
DCZ-TTR ^(^^b),m^	3.2	~5000	---	---	59.6	---	20.1	512	[34]
CZ-TTR ^(^^b),(^^c),m^	3.1	~1000	---	---	32.5	---	14.4	492	[34]
Dev. 2 *^,d^	---	---	---	---	---	---	4.71	431	[52]
BITPI *^,m^	2.6	---	---	---	6.7	---	7.1	452	[53]
BITPI *^,s^	3.1	---	---	---	3.4	---	2.98	452	[53]

^†^ Here are shown the best values, for deviations/error bars (for OLEDs based on CZ-2 and CZ-1) see Figure S8 (SI). EML = Emissive Layer, (a) carbazole emitter (EML), (b) carbazole host/guest, (c) wavelength similar to our devices, * devices fabricated by evaporation. V_on_ = turn on voltage, L_max_ = maximum luminance, V_Lmax_ = voltage for L_max_, ƞ_Lmax_ = current efficiency at L_max_, ƞ_max_ = maximum current efficiency, Lƞ_max_ = luminance at ƞ_max_, EQE_max_ = maximum external quantum efficiency, λ_EL_ = peak of electroluminescence. s = single layer device, d = double layer device, m = multilayer device.

## Supplementary Materials

The supplementary materials are available online.
