# Supplementary material for: Efficient OLEDs Fabricated by Solution Process Based on Carbazole and Thienopyrrolediones Derivatives
Source: Molecules. 2018 Jan 30;23(2):280. doi: 10.3390/molecules23020280 (PMC6017460; doi:10.3390/molecules23020280)
Supplement: Supplementary file 1 [file molecules-23-00280-s001.pdf]

Supplementary Information

Efficient OLEDs fabricated by solution process based on carbazole and thienopyrrolediones derivatives

L. A. Lozano-Hernández [1], J. L. Maldonado [1,*], C. Garcias-Morales [1,3], A. E. Roa [1,†], O. Barbosa-García [1], M. Rodríguez [1] and E. Pérez-Gutiérrez [2]

[1] Research Group of Optical Properties of Materials (GPOM), Centro de Investigaciones en Óptica, A.P. 1-948, 37000 León, Guanajuato, México; luislozano@cio.mx (L.A.L.-H.); jlmr@cio.mx (J.L.M.); cgarcias@uadec.edu.mx (C.G.-M); arian.espinosa@ciqa.edu.mx (A.E.R.); barbosag@cio.mx (O.B.-G.); mrodri@cio.mx (M.R.)

[2] CONACYT-Laboratorio de Polímeros, Centro de Química, Instituto de Ciencias, Benemérita Universidad Autónoma de Puebla (BUAP), Complejo de Ciencias, ICUAP, Edif. 103H, 22 Sur y San Claudio, C.P. 72570 Puebla, Puebla, México; eperez@conacyt.mx (E.P.-G.)

[3] Departamento de Química Orgánica, Facultad de Ciencias Químicas, Universidad Autónoma de Coahuila, Saltillo, 25280, Coahuila, México.

* Correspondence: jlmr@cio.mx (J.L.M.); Tel.: +52-477-441-4200 (ext. 265) (J.L.M.)

[†] Present address: CONACYT-Centro de Investigación en Química Aplicada, Unidad Monterrey, Alianza Sur No. 204 Parque de Innovación e Investigación Tecnológica (PIIT), Apodaca, 66600 Nuevo León, México.

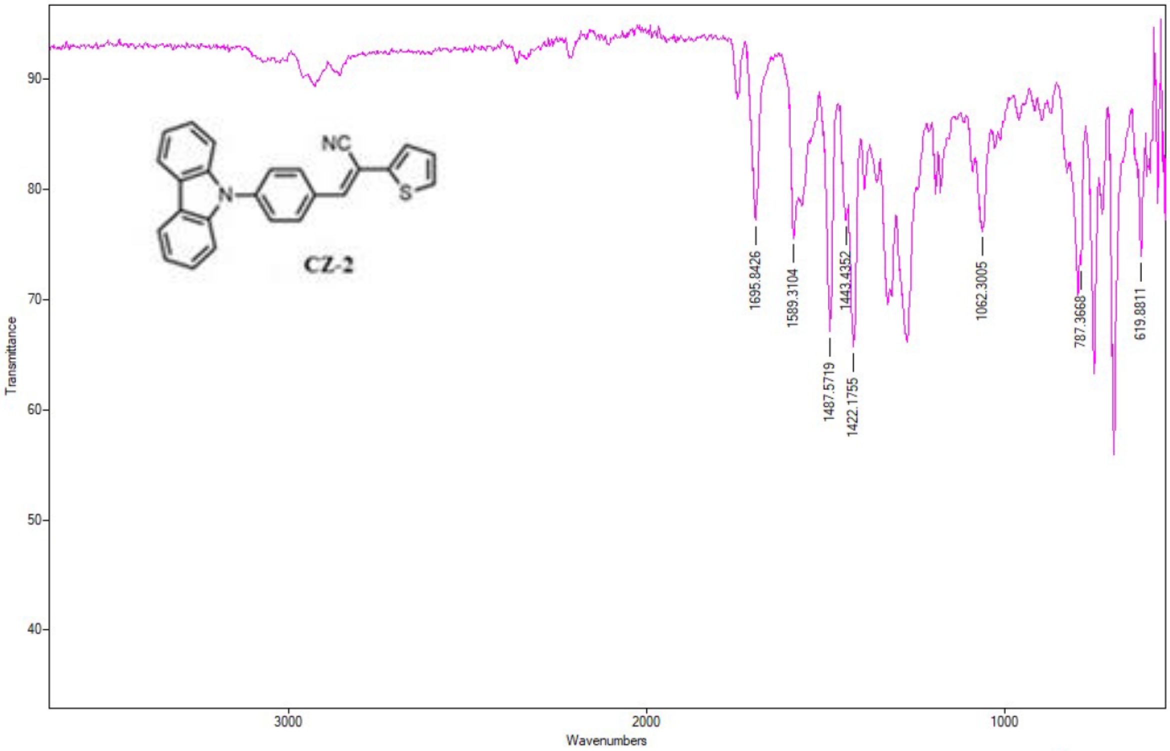

Supplementary Figure S1. IR of molecule **CZ-2**.

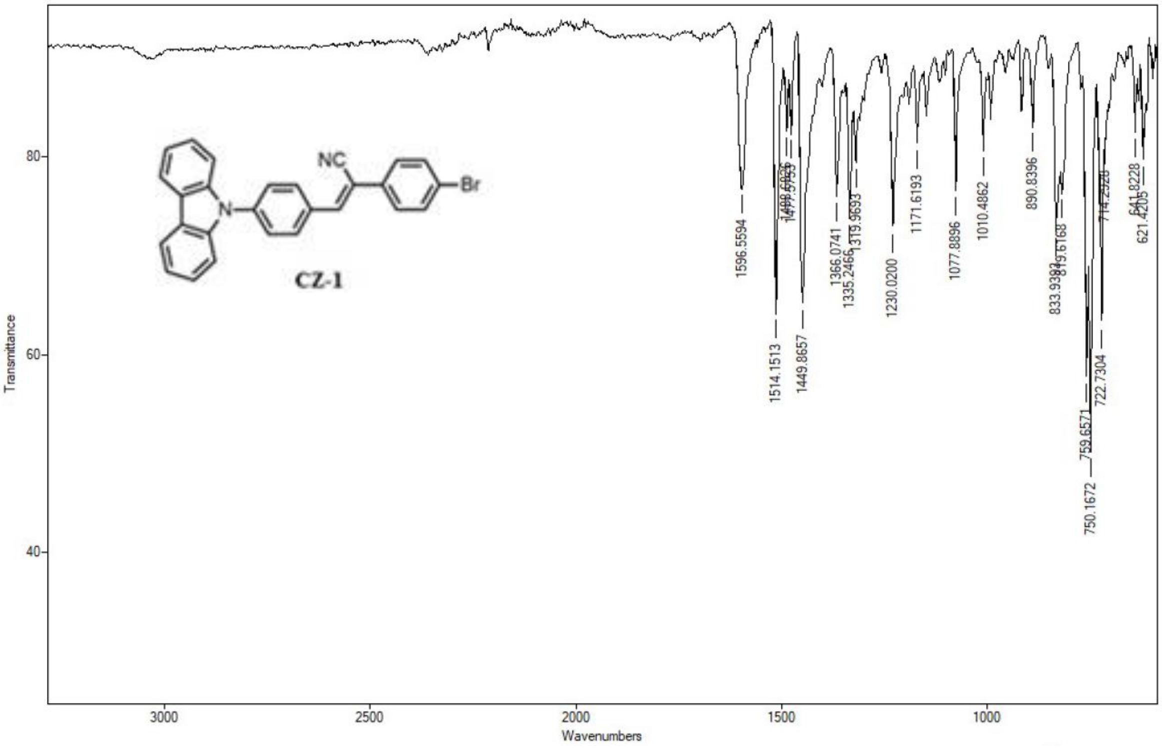

Supplementary Figure S2. IR of molecule **CZ-1**.

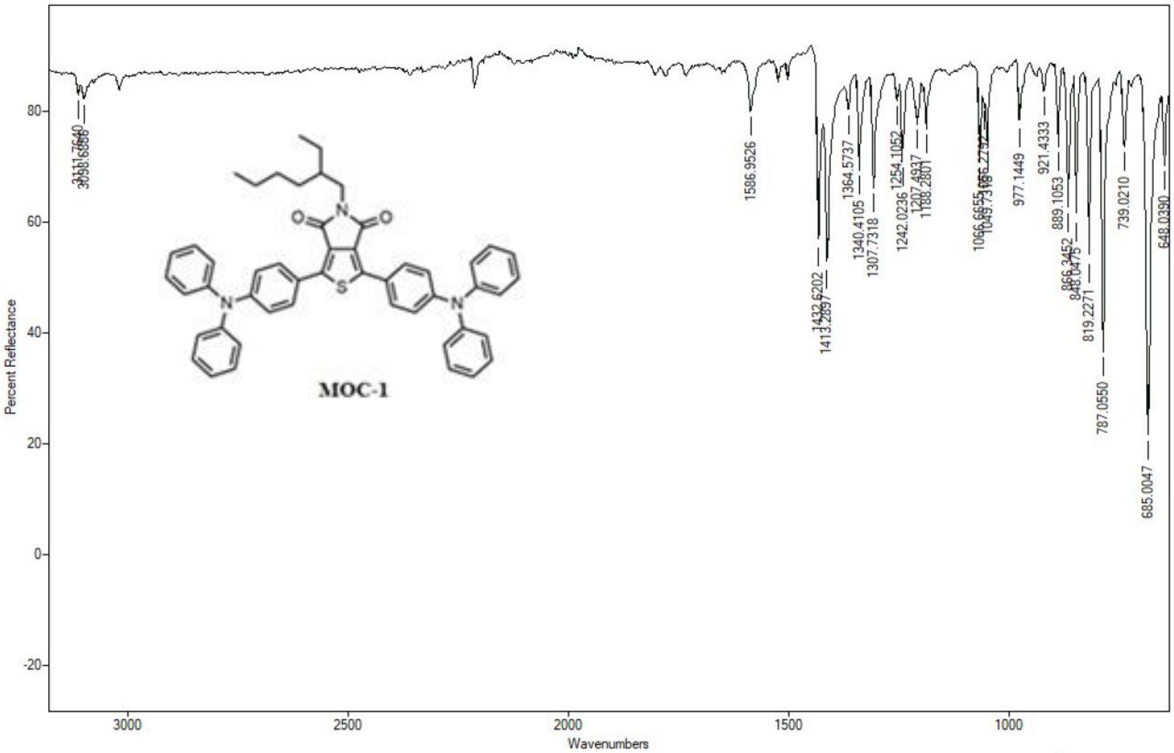

Supplementary Figure S3. IR of molecule **MOC-1**.

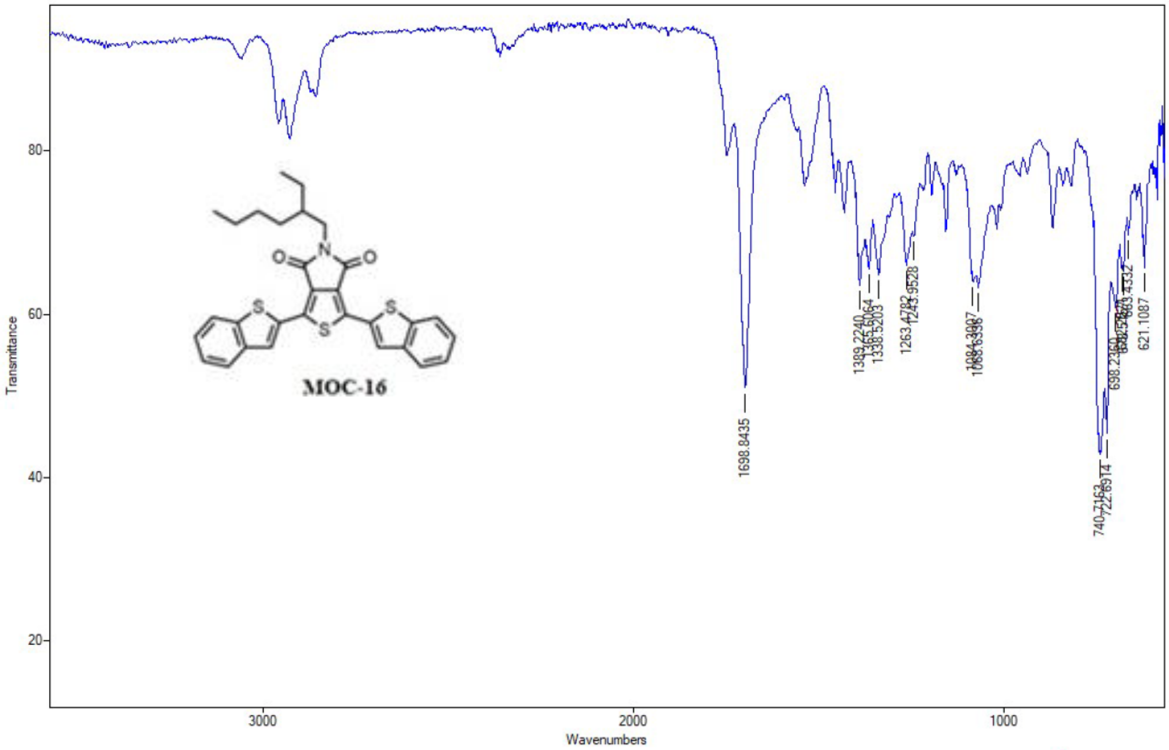

Supplementary Figure S4. IR of molecule **MOC-16**.

Quantum yield (QY) of **CZ-1** molecule (in solid state film) and the fluorescence lifetime (concentration of 1.11 × 10^{-5} mol/L) were also measured using a spectrofluorometer (Edinburgh instruments, FS5 spectrofluorometer).

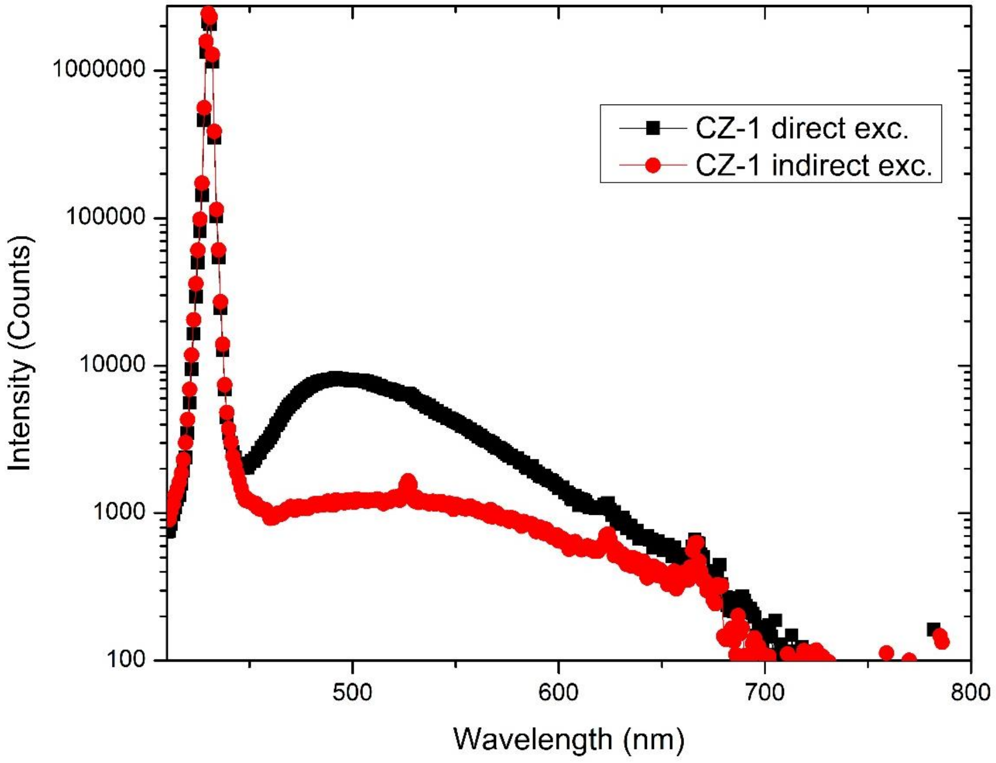

Supplementary Figure S5. Intensities with direct exc. and indirect exc. measured for **CZ-1** molecule in solid sate film. PLQY = 51.9%.

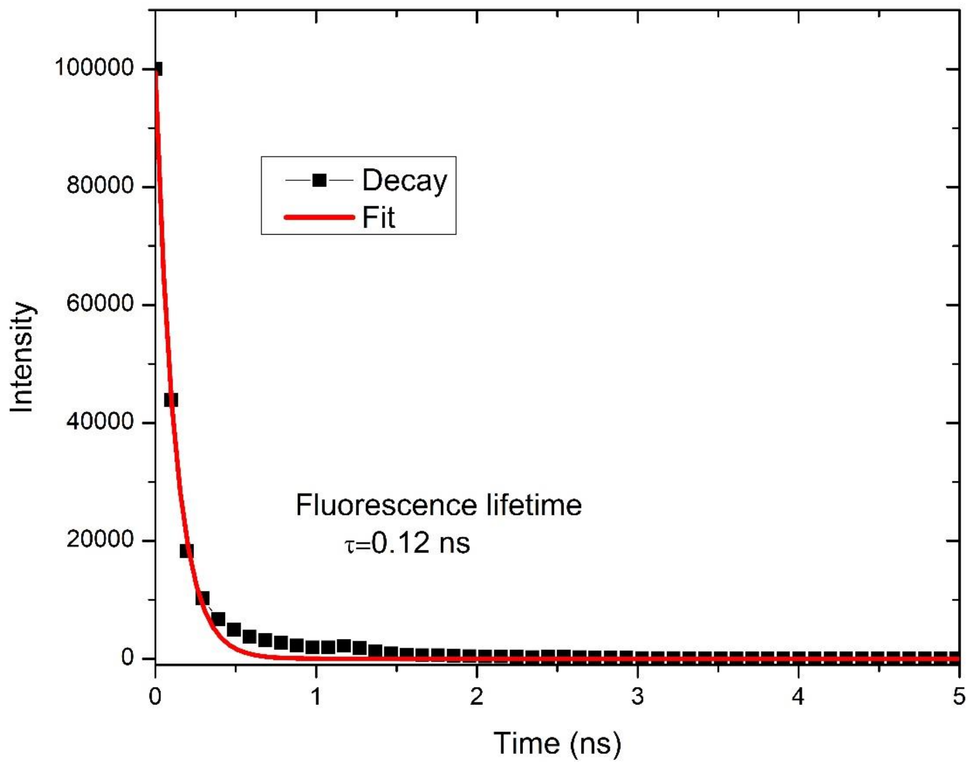

Supplementary Figure S6. Fluorescence lifetime for **CZ-1** molecule in solution.

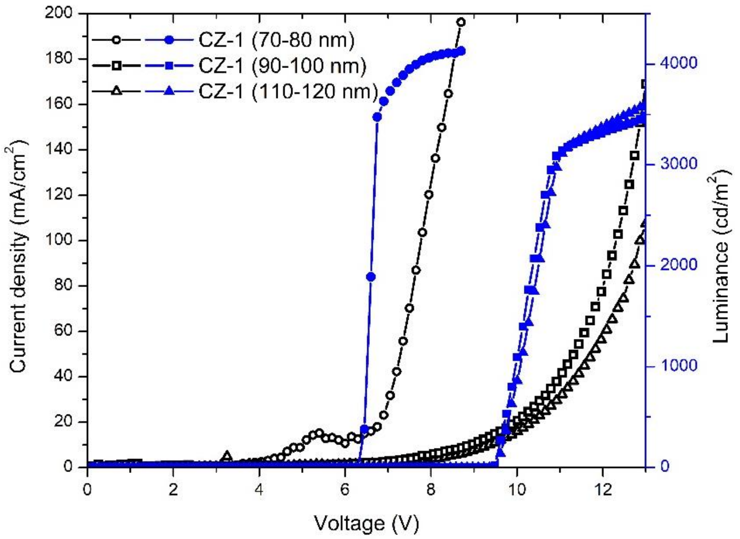

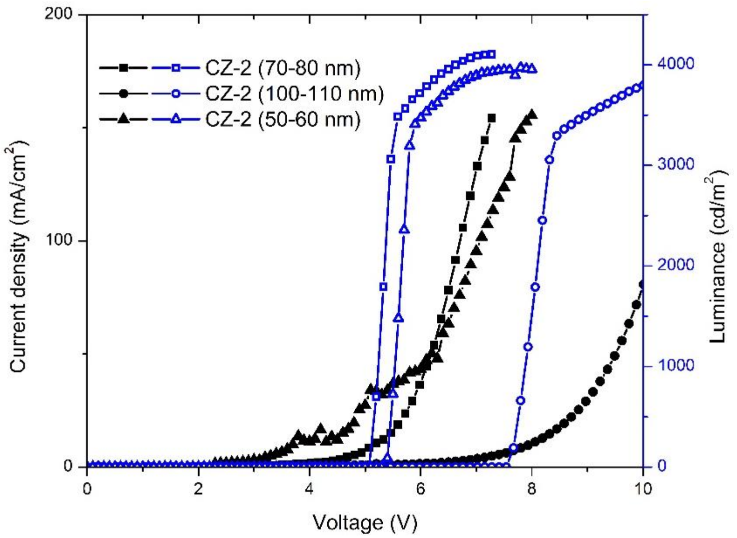

Supplementary Figure S7. J-L-V curves for the best 3 OLEDs for three different thicknesses with EMLs based on CZ-1 and CZ-2.

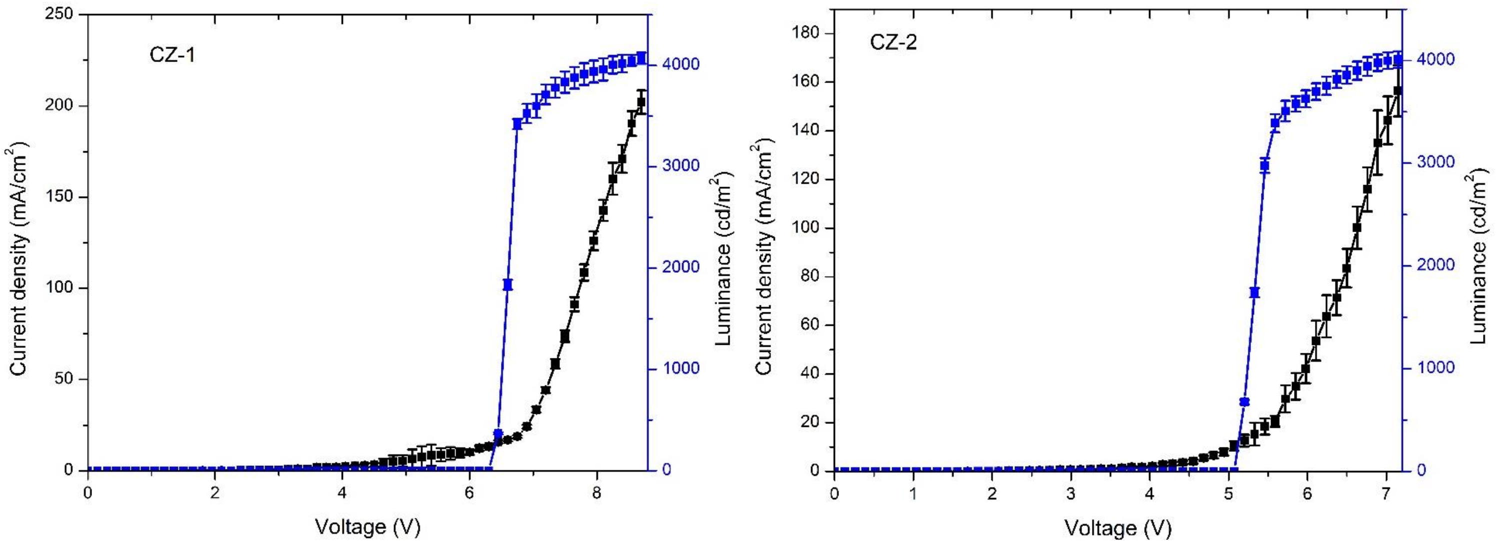

Supplementary Figure S8. Average J-L-V curves with standar deviation/errors bars for a set of three different fabricated OLED devices (for each EML), with EMLs based on **CZ-1** and **CZ-2** (EML thickness: 70-80 nm)
